# Valorisation of banana stem into N-doped activated carbon as a selective sorbent for cationic dyes and pharmaceutical contaminants

**DOI:** 10.1039/d5ra09071g

**Published:** 2026-02-27

**Authors:** Alibasha Akbar, M. Bhavani Lakshmi, Priyadip Das, Quazi Arif Islam, Paramita Pattanayak, Tanmay Chatterjee, Sritama Mukherjee, Mihir Ghosh

**Affiliations:** a Department of Chemistry, SRM Institute of Science and Technology, Kattankulathur (SRMIST-KTR) Tamil Nadu 603203 India mihirg@srmist.edu.in; b Department of Chemistry, Alipurduar University P.O.-Alipurduar Court Alipurduar West Bengal 736122 India; c Department of Chemistry, Birla Institute of Technology and Science, Pilani (BITS Pilani), Hyderabad Campus Jawahar Nagar, Kapra Mandal Hyderabad 500078 India; d Division of Fiber and Polymer Technology, CBH, KTH Royal Institute of Technology Teknikringen 56-58 SE-100 44 Stockholm Sweden

## Abstract

The persistent discharge of synthetic cationic dyes and pharmaceutical residues into aquatic environments necessitates the development of sustainable, high-performance sorbents for wastewater treatment. This study presents the design and synthesis of porous nitrogen-doped activated carbon (PNAC) derived from banana plant stems, demonstrating its excellent potential as a bio-based adsorbent for wastewater purification. PNAC exhibits a remarkably high surface area of 1978.8 m^2^ g^−1^ and abundant nitrogen functionalities that collectively enhance adsorption capacity, selectivity, and kinetics. It achieves over 95% removal of methylene blue, brilliant green, and crystal violet within 20 minutes, outperforming commercial activated carbon (84%, 54%, and 76% removal, respectively). The adsorption process follows the Langmuir isotherm model, confirming monolayer coverage, and proceeds through hybrid pseudo-second-order and intraparticle diffusion mechanisms. Thermodynamic analysis reveals a spontaneous and endothermic adsorption nature, indicating strong interactions between PNAC and the pollutant molecules. Beyond synthetic dye removal, PNAC also exhibits efficient uptake of pharmaceutical contaminants, 79.8% for ciprofloxacin and 81.1% for cefixime, within 30 minutes and achieves 97% dye removal from real textile effluents within 15 minutes. The material demonstrates excellent recyclability, retaining approximately 80% efficiency after five adsorption–desorption cycles and 85% after eight cycles of acid-assisted regenerations. These findings highlight PNAC as a scalable, eco-friendly, and high-performance sorbent derived from agricultural waste, offering a promising platform for next-generation wastewater treatment and sustainable environmental remediation.

## Abstract 2

The persistent discharge of synthetic cationic dyes and pharmaceutical residues into aquatic environments necessitates the development of sustainable, high-performance sorbents for wastewater treatment.

## Graphical abstract

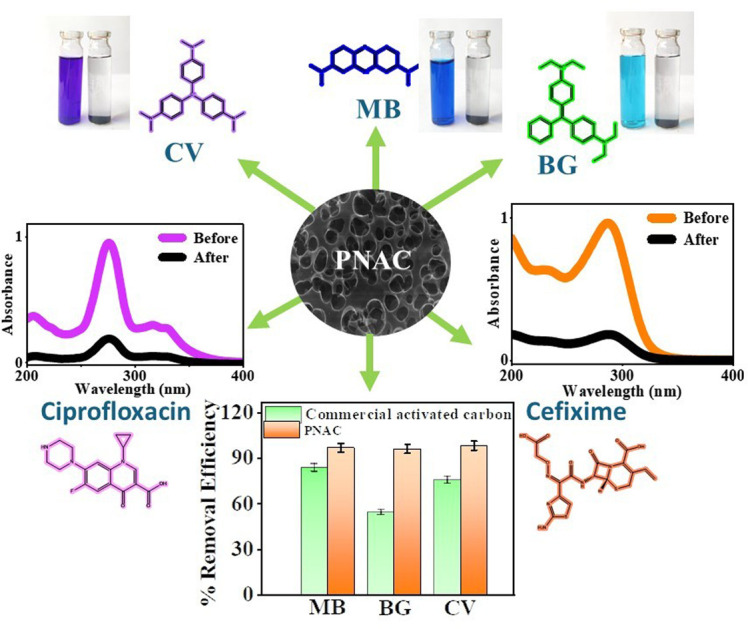

## 1. Introduction

The availability of clean water is fundamental to human health, sustainable development, and ecological stability. Yet, freshwater resources are under mounting pressure from rapid urbanisation, industrialisation, agricultural intensification, and climate change.^1,2^ A critical outcome has been the deterioration of water quality through continuous discharge of chemical and biological pollutants into aquatic systems. Among these, synthetic organic dyes and pharmaceutical residues represent two particularly persistent and environmentally hazardous pollutant classes.^3,4^ The global production of synthetic dyes is approximately 1 million tonnes.^5^ Of this total, 1–2% (∼10–20 thousand tonnes) are synthetic cationic dyes as used in this study. Their structural complexity, high chemical stability, and resistance to degradation render them recalcitrant to conventional wastewater treatment methods such as coagulation, flocculation, and biological oxidation.^6–9^ Cationic dyes, including methylene blue (MB), brilliant green (BG), and crystal violet (CV), were selected as well-characterized model compounds in this study. They serve as standardized molecular probes to systematically evaluate the intrinsic adsorption properties and surface chemistry of the developed sorbent under controlled conditions, providing a reliable benchmark for performance comparison. Their strong chromophores impede aquatic photosynthesis, while their mutagenic and carcinogenic properties pose significant toxicological risks to humans and ecosystems.^10,11^ Similarly, pharmaceutical residues continuously released into surface and groundwaters through municipal and industrial effluents exhibit bioactive persistence, ecological toxicity, and potential to induce antimicrobial resistance.^12^ The coexistence of such pollutants in industrial effluents and natural waters underscores the need for effective, selective, and sustainable remediation strategies. Physicochemical technologies, including advanced oxidation processes, photocatalysis, electrochemical treatments, and membrane filtration, have been widely explored for dye and pharmaceutical removal.^13–16^ Although these methods can achieve high efficiencies under controlled conditions, they are limited by high energy consumption, operational complexity, and secondary waste generation. In this context, adsorption has emerged as an attractive alternative, offering broad-spectrum applicability, modular design, scalability, and low operational cost.^17,18^ Activated carbon is the benchmark adsorbent owing to its large surface area, developed porosity, and tunable surface chemistry that enables electrostatic interaction, π–π stacking, hydrogen bonding, and van der Waals forces with organic pollutants. However, the widespread use of commercial activated carbon is restricted by its high production costs and reliance on non-renewable precursors.^19–22^

Therefore, the development of bio-derived carbon from agricultural wastes offers a sustainable strategy that aligns with both circular economy principles and water remediation needs.^23,24^ In this scenario, banana stems, an abundant lignocellulosic residue generated in tropical and subtropical regions, represent a promising precursor due to its high carbon content, low cost, and ease of processing. The activity of bio-derived carbon can be further enhanced by different modifications. Surface modification further enhances the sorption performance of biomass-derived carbons.^25^ Nitrogen doping introduces electron-donating heteroatoms and polar functionalities (pyridinic, pyrrolic, and graphitic nitrogen), improving affinity for cationic dyes and pharmaceuticals.^26^ Coupled with KOH activation, which generates hierarchical porosity and significantly enlarges the accessible surface area, a synergistic enhancement in adsorption efficiency is achieved.^27,28^ Despite these advances, studies integrating nitrogen doping with alkali activation in biomass-derived carbons for the simultaneous removal of structurally diverse pollutants such as cationic dyes and pharmaceuticals under industrial wastewater conditions remain limited.

Addressing this research gap, the present work aims to valorise banana stem biomass into hierarchical porous nitrogen-doped activated carbon (PNAC) *via* a green synthesis route. The study investigates the efficacy and mechanisms of selective adsorption of cationic dyes, pharmaceutical contaminants and real textile effluent, providing new insights into the design of sustainable sorbents for large-scale water purification applications.

## 2. Experimental section

### 2.1 Chemicals and reagents

The banana stems (*Musa acuminata* stems) were obtained from a nearby local village field and were the primary source for producing activated carbon. Urea (Loba Chemie, ≥99%, India), potassium dihydrogen orthophosphate (SRL Chemicals, ≥99.5%, India), potassium phosphate dibasic anhydrous (SRL Chemicals, ≥99.5%, India), sodium hydroxide (SRL Chemicals, ≥97%, India), potassium hydroxide powder 80–100 mesh (SRL Chemicals, ≥85%, India), hydrochloric acid extra pure (Loba Chemie, 37%, India), methylene blue (C.I. Basic Blue 9, SRL Chemicals, India), crystal violet (C.I. Basic Violet 3, SRL Chemicals, India), and brilliant green (C.I. Basic Green 1, SRL Chemicals, India), safranin O (C.I. Basic Red 2, SRL Chemicals, India), eosin B (C.I. Acid Red 91, SRL Chemicals, India), direct red 81 (C.I. Direct red 81, SRL Chemicals, India), methyl orange (C.I. Acid Orange 52, SRL Chemicals, India), congo red (C.I. Direct Red 28, SRL Chemicals, India), charcoal activated (280) ExiPlus (SRL Chemicals, India), were used directly without further purification. A one-gram amount of each dye was weighed and dissolved in 1000 mL of deionized water to prepare a standard dye solution of 1000 mg L^−1^ as a stock solution. For the adsorption test experiments, different concentrations of dye solution were prepared using the stock solution by diluting it with deionized water. The pH influence was analyzed using phosphate buffer solutions (PBS), prepared with phosphate salts, and adjusted with 0.1 M NaOH or 0.1 M HCl.

### 2.2 Preparation of nitrogen-doped porous carbon from *Musa acuminata* stem

**Scheme 1:**
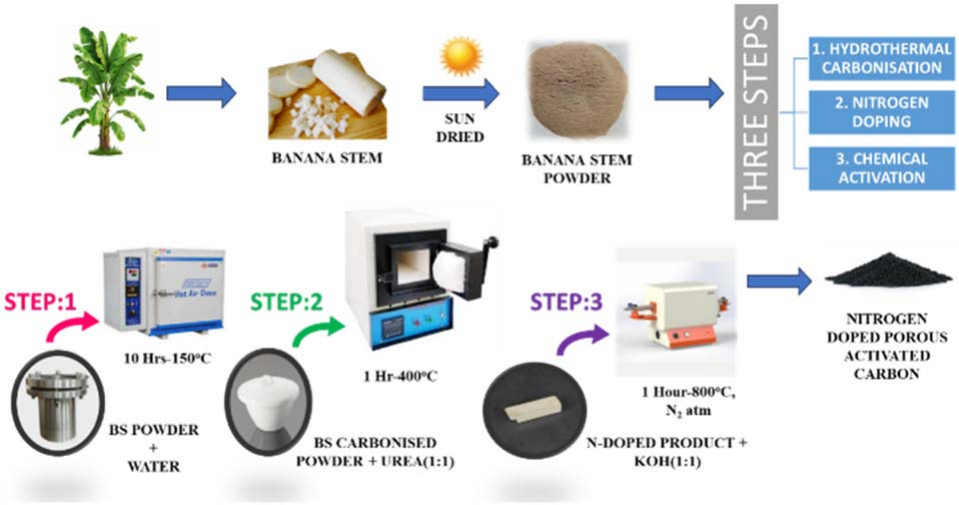
Pictorial representation of the preparation procedure of PNAC adsorbent from banana stem biomass.

The collected banana stems were initially washed, peeled, sliced, sun-dried, and ground into fine powder to serve as the biomass precursor. After the sun drying the average weight loss content was ∼94.7%. The nitrogen-doped porous activated carbon (PNAC) was synthesized through a three-step process involving hydrothermal carbonization, nitrogen doping, and chemical activation. In the first step, 7 g of banana stem powder was dispersed in 70 mL of distilled water in a 100 mL Teflon-lined autoclave and subjected to hydrothermal treatment at 150 °C for 10 hours. The resulting hydrochar was recovered, repeatedly washed with distilled water, and oven-dried at 100 °C. For nitrogen incorporation, an equal mass (1 : 1 w/w) mixture of hydrochar and urea was calcined in a muffle furnace at 400 °C for 1 hour in an air atmosphere, yielding N-doped carbon. In the final step, activation and pore development were achieved by mixing the N-doped carbon with KOH (1 : 1 w/w), followed by thermal treatment in a tubular furnace under N_2_ flow for 1 hour. Activation temperatures of 400, 550, 700, and 800 °C (heating rate: 10 °C min^−1^) were employed, generating a series of samples designated PNAC-400, PNAC-550, PNAC-700, and PNAC-800. Post-activation, the products were thoroughly washed with 0.01 M HCl and distilled water until a neutral pH was achieved, and then oven-dried at 80 °C for 12 hours. The resulting PNACs were characterized in detail, and adsorption experiments were conducted against three model cationic dyes (MB, CV and BG), pharmaceutical contaminants and real textile effluent. The overall synthesis pathway is schematically depicted in Scheme 1.

### 2.3 Characterizations

The phase analysis of the samples was assessed using a Panalytical Xpert Pro X-ray diffractometer with data collection ranging from 2*θ* = 10° to 80° and an increment of 0.025° with Cu Kα line with a wavelength of 0.154 nm. A Fourier-transform infrared spectroscopy (FTIR) study was carried out using a Tensor 27 spectrophotometer (Bruker, Bremen, Germany) to analyze the chemical structure of hierarchical porous N-doped carbon across the range of 4000–400 cm^−1^. Nitrogen physical adsorption experiments were performed using Microtrac Bel-BEL SORP mini II model analyzer at BITS Pilani, Hyderabad to determine the surface area, pore volume, and pore diameter of the samples by the Brunauer–Emmett–Teller (BET) method. The morphology of the samples was investigated using scanning electron microscopy (Bruker HR-SEM (Thermo Scientific Apreo S)) in conjunction with energy dispersive X-ray spectroscopy (EDX). High-resolution transmission electron microscopy (HRTEM) was performed using a JEOL 2010F TEM at an accelerating voltage of 200 kV. X-ray photoelectron spectroscopy (PHI Versa Probe III) with Al Kα radiation as the excitation source was used to determine the chemical state of the elements. Adsorption experiments of dyes using PNAC were done by using Shimadzu (UV-19001) dual-beam UV-visible spectrophotometer.

### 2.4 Dye adsorption study

Batch adsorption experiments were systematically conducted to evaluate the removal efficiency of PNAC-800 toward cationic dyes, MB, BG, and CV, under a wide range of operational parameters including pH (2–12), initial dye concentration (20–200 mg L^−1^), contact time (1–20 minutes), adsorbent dosage (0.1–1 g), and temperature (30–60 °C). Stock dye solutions (1000 mg L^−1^) were first prepared and subsequently diluted to obtain working solutions, 100 mg L^−1^ for BG, and 80 mg L^−1^ for MB and CV. For each adsorption run, a predetermined mass of PNAC-800 (0.6 g for MB; 0.7 g for BG and CV) was introduced into Erlenmeyer flasks containing the dye solutions. The suspensions were agitated using a mechanical shaker at 100 rpm, and equilibrium was typically achieved within 20 minutes. At regular intervals, 2 mL aliquots were withdrawn and analyzed by UV-vis spectroscopy until the absorbance reached zero, confirming complete dye removal.

The percentage of removal and adsorption capacities of dyes by PNAC-800 were calculated by using the following equations:1
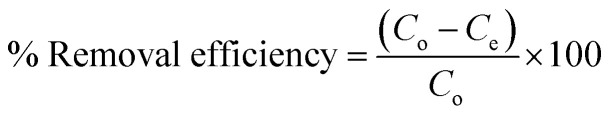
where, *C*_o_ is the initial dye concentration (mg L^−1^), *C*_e_ is the equilibrium concentration of the dye (mg L^−1^), and % removal is the percentage of the dye removed.2
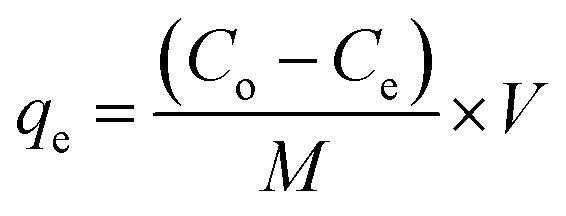
Here, *q*_e_ is the adsorbed quantity of dye (amount of dye adsorbed per unit mass of adsorbent), *C*_o_ is the initial dye concentration in the solution, *C*_e_ is the equilibrium concentration of the dye in the solution (after adsorption), *M* is the adsorbent mass, and *V* is the volume of the solution. For the reusability study, the adsorbent was separated from the reaction mixture by centrifugation (10 000 rpm, 5 min), washed three times with distilled water, and dried at 80 °C for 2 hours. The regenerated material (0.6 g/0.7 g) was subsequently reused for adsorption under identical conditions. After each cycle, any loss in mass was compensated by supplementing with the minimum amount of pre-used adsorbent collected from parallel runs. This procedure was repeated to evaluate the recyclability of the material.

## 3. Results and discussion

### 3.1 Characterizations of synthesized materials

**Fig. 1:**
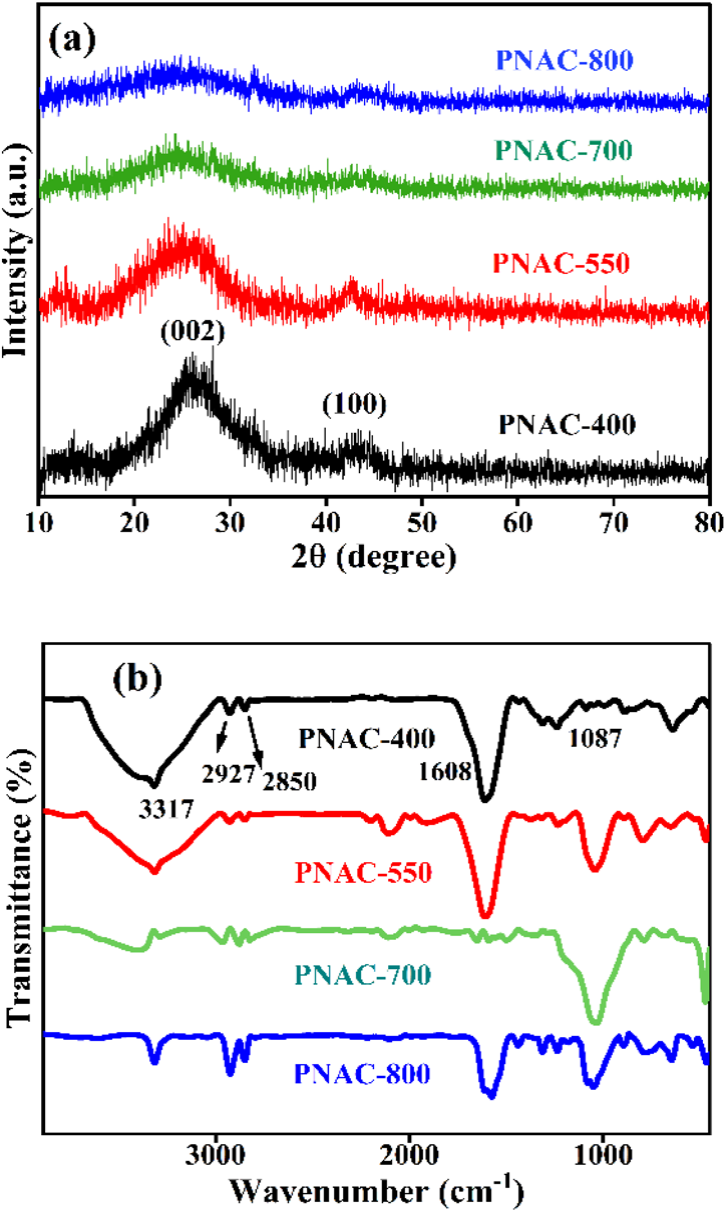
PNAC with different pyrolysis temperatures in N_2_ atmosphere (a) XRD spectra and (b) FT-IR spectra.

The XRD patterns of the synthesized PNAC materials at different temperatures are shown in Fig. 1a. It reveals important information about their crystalline structure and phase composition. Two prominent diffraction peaks are observed at approximately 26.5° and 42.6° across all samples, corresponding to the partially graphitic carbon.^29^ The broadness of all the peaks, along with the lack of characteristic signals of highly graphitized carbon, suggests that the degree of graphitization is low and amorphous carbon is formed. It is also observed that the degree of graphitization decreases slightly as the activation temperature increases from 400 °C to 800 °C. This may be attributed to the N-doping in the carbon, which improves the disorder in the structure.^30^ During the preparation process, the interaction of urea and KOH on the surface forms defect structures that destroy the partial crystalline graphitic structure, which is enhanced with the temperature rise. The spectra in Fig. 1b reveal a broad O–H stretching band at 3317 cm^−1^ for PNAC samples, indicative of hydrogen-bonded hydroxyl groups in the hydrophilic carbon matrix. The bands at around 2927 cm^−1^ and 2850 cm^−1^ are associated with C–H stretching vibrations of methylene (CH_2_) groups. Additionally, a peak at approximately 1608 cm^−1^ is assigned to C–N stretching^31^ and the band at 1087 cm^−1^ is attributed to the phenolic C–O stretching vibration.^32^ The intensities of the peaks decrease at higher pyrolysis temperatures due to thermal decomposition.^33^ In comparison, the BS hydrochar spectrum (Fig. S1a) shows analogous features shifted slightly (*e.g.*, O–H at 3361 cm^−1^, C–H at 2916 cm^−1^ and 2852 cm^−1^, C=O at 1635 cm^−1^), reflecting preserved oxygen functionalities on post-hydrothermal treatment. Thermogravimetric analysis of BS hydrochar shown in Fig. S1b reveals three distinct weight loss stages up to 1000 °C, reflecting its thermal decomposition profile. Initial approximately 13.64% loss below 240 °C corresponds to desorption of physisorbed water and low-boiling compounds volatiles from hydrophilic surface groups. A major devolatilization stage (∼68.3% loss) between 200–600 °C involves decomposition of hemicellulose, cellulose, and labile oxygen functionalities, consistent with hydrochar's biomass-derived structure. Final char residue stabilizes at ∼24.53% above 600 °C, mainly graphitic carbon and mineral ash resistant to further oxidation.

Raman spectroscopy was performed to evaluate the degree of structural deformation in the synthesised PNAC carbon materials, and the results are shown in Fig. S1c. It exhibits two broad prominent peaks at 1320 cm^−1^ and 1560 cm^−1^. The first peak, the D band, indicates a disordered structure. This forbidden mode turns active due to the lack of long-range order in amorphous and quasi-crystalline forms of carbon materials.^34^ The intensity of this band may also increase due to imperfections like sp^3^ carbon, N doping, and small graphitic domains.^34^ On the other hand, the G band (at 1560 cm^−1^) corresponds to a crystalline graphite structure with a sp^2^ hybridized carbon atom. The width of this band is also an indicator of the overall crystallinity of a carbon material. The broader width indicates a more amorphous nature of the sample.^35^ The *I*_D_/*I*_G_ intensity ratio can reflect the level of graphitisation in carbon materials and their structural disorder. In the present research, prepared samples give very close values for *I*_D_/*I*_G_ of 0.85 for PNAC-400 and 0.84 for PNAC-550, 700, and 800. The values, along with the broad nature of the peaks, indicate the amorphous nature of the samples with partial graphitization containing structural defects caused by nitrogen atom incorporation and the activation process as discussed earlier.^30^ The findings of the Raman study correlate well with the XRD data.

**Fig. 2:**
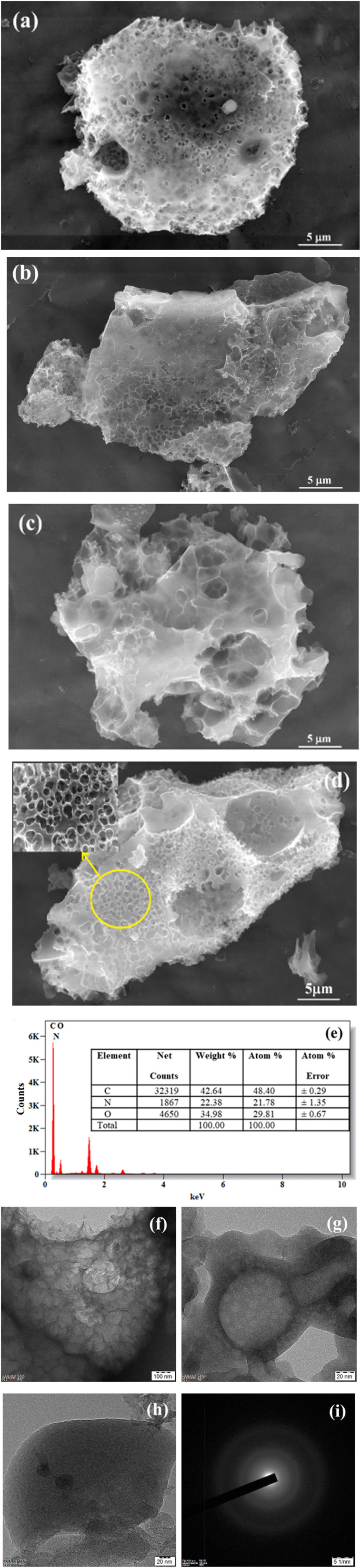
HRSEM images of PNAC synthesized from the banana stem at different pyrolysis temperatures in N_2_ atmosphere (a) PNAC-400, (b) PNAC-550, (c) PNAC-700, (d) PNAC-800. Additionally, (e) shows the EDX spectra of the PNAC-800 sample. TEM images of the PNAC-800 material are displayed in different magnifications in (f–h), while (i) features the SAED pattern of the PNAC-800 sample.

HRSEM images of the synthesized PNAC at varying pyrolysis temperatures (400 °C to 800 °C) reveal a consistently rough and porous surface morphology, as illustrated in Fig. 2a–d. Notably, there is a progressive increase in pore size and uniformity with higher pyrolysis temperatures. Specifically, Fig. 2a depicts PNAC-400, which exhibits minimal pore formation, with only slight initiation of porosity at 400 °C. As the temperature increases to 550 °C and 700 °C, as shown in Fig. 2b and c for PNAC-550 and PNAC-700 respectively, a marked enhancement in pore structure is observed. Fig. 2d for PNAC-800 reveals a notable enhancement in both porosity and the uniformity of pore distribution. The adsorbent exhibits a hierarchical porous structure developed during pyrolysis at 800 °C. These pores are not only present on the surface but are also prominently integrated within the inner matrix of the adsorbent, contributing to its overall structural integrity and adsorption capacity. The HRSEM analysis indicates that elevated pyrolysis temperatures facilitate increased pore size and density. The heightened porosity at these temperatures can be attributed to the thermal degradation of organic contaminants and subsequent activation processes, which promote the formation of more interconnected porous networks. This enhanced porosity is particularly advantageous for adsorption applications, as it maximizes the available surface area for interaction with dye molecules. The highly porous architecture allows efficient adsorption, providing numerous active sites and promoting enhanced diffusion of dye molecules into the internal pores. Fig. 2e presents the EDX spectrum of PNAC-800 along with its elemental composition table, confirming the presence of the expected elements in the material. Fig. 2f–i shows the various magnifications of TEM images and the selected area electron diffraction (SAED) pattern of PNAC-800. Fig. 2f–h illustrate the surface porosity of PNAC-800, revealing a well-developed hierarchical porous architecture within its network. The carbon exhibits an amorphous nature, although some crystallinity is evident in the electron diffraction pattern, as shown in Fig. 2i. This supports the findings from XRD, Raman, and SEM analyses.

**Fig. 3:**
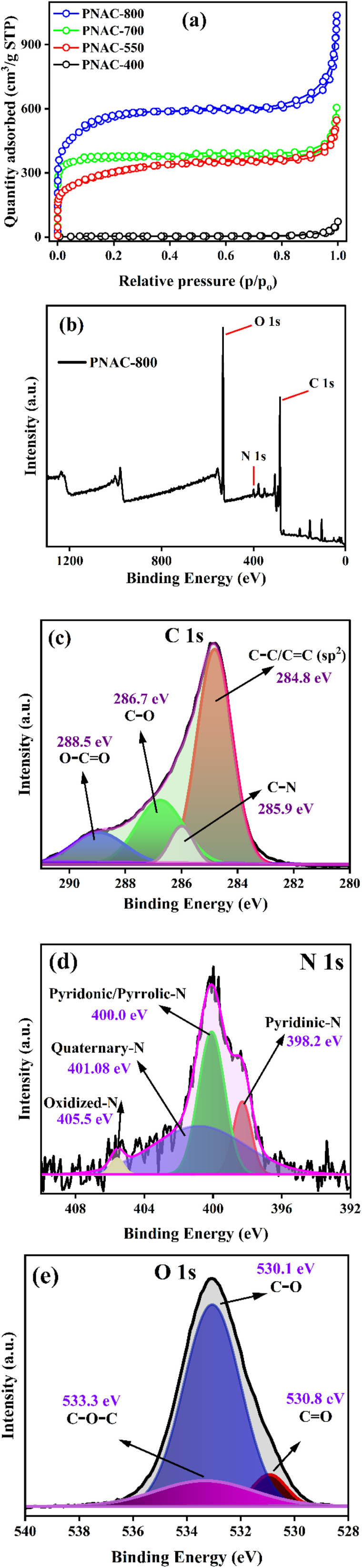
(a) BET isotherm plots of PNAC-400, 550, 700, and 800 samples. XPS spectra of PNAC-800: (b) survey spectra, (c) C 1s core level spectra, (d) N 1s core level spectra, and (e) O 1s core level spectra.

The porous structure of PNAC materials is further examined through N_2_ sorption isotherms at 77 K. Fig. 3a shows the BET isotherm analysis of PNACs, which exhibited a type IV behaviour with a H3 hysteresis loop according to the IUPAC classification, indicating the presence of micro–mesoporous structure.^36^ The steep increase at low relative pressure (*P*/*P*_0_ < 0.1) suggests the presence of micropores, while a relative pressure of 0.1–0.9 reveals the existence of mesopores. The tails at a relative pressure of 1.0 indicated the presence of limited macropores, confirming the hierarchical pore structures of PNACs.^37,38^ BET analysis reveals that the PNAC-800 sample has a higher surface area and pore volume compared to the PNAC-400, 550, and 700 samples. Specifically, the measured surface area of PNAC-800 is 1978.8 m^2^ g^−1^, with a total pore volume of 1.35 cm^3^ g^−1^. The surface area and pore volume of PNAC-700, 550, and 400 samples are found to be 1442.9 m^2^ g^−1^, 1046.1 m^2^ g^−1^, and 11.069 m^2^ g^−1^ and 0.80 cm^3^ g^−1^, 0.76 cm^3^ g^−1^, and 0.06 cm^3^ g^−1^ respectively. The findings demonstrate that PNAC-800 displays a combination of microporosity and mesoporosity, with a markedly elevated specific surface area and pore volume compared to the other samples.

Fig. 3b shows the XPS survey spectra of PNAC-800 adsorbent material showing C 1s, O 1s, and N 1s. The XPS technique is used to assess the chemical state and primary composition of the elements present in the sample. This method provides in-depth and significant insights into how KOH chemical activation influences the surface characteristics of the activated carbon. The asymmetric C 1s spectra can be separated into four distinct peaks, shown in Fig. 3c. The peak around 284.8 eV corresponds to C–C (or) C=C bonds, while a peak at 285.9 eV is associated with C–N. The peak at 286.7 eV is related to C–O, and the peak at 288.5 eV refers to O–C=O.^39,40^ XPS peak differentiation for the N 1s region was conducted to identify various nitrogen functionalities in PNAC-800, as illustrated in Fig. 3d. The XPS spectrum exhibits a broad signal centred at 400.1 eV, which was deconvoluted into four distinct components. The peak at 398.2 eV corresponds to pyridinic-N, while the peak near 400.0 eV is assigned to overlapping contributions from pyridinic-N and pyrrolic-N species. The signal at 401.08 eV is associated with quaternary-N, and the higher binding energy peak at 405.5 eV indicates the presence of oxidized-N functionalities in the N-doped activated carbon.^41^ These nitrogen-containing groups play a crucial role in enhancing the dye adsorption capacity of the material by providing active surface sites that facilitate electrostatic attraction with dye molecules. Furthermore, n–π interactions between the aromatic domains of the dyes and the graphitic structure of PNAC-800 strengthen the overall adsorption process carbon.^42,43^ Oxygen-containing functional groups like ethers, carbonyls, and hydroxyls enhance the adsorptive capacity of carbon materials.^44,45^ The high-resolution O 1s spectra for PNAC-800, shown in Fig. 3e, reveal three peaks-a carbonyl group (–C=O) at 530.8 eV, an ether group (C–O–C) at 533.3 eV, and a hydroxyl group (–C–OH) at 530.1 eV.^40,46^ The XPS spectra of PNAC-400 are shown in Fig. S2a–d and compared to those of the PNAC-800 sample. The PNAC-800 sample demonstrates a greater abundance of functional groups, highlighting the influence of temperature during the preparation of activated carbon for dye adsorption. To further analyze the surface, zeta potential measurements have been carried out. Fig. S3a and b shows the details of the zeta potential measurements for the adsorbents PNAC-400 and PNAC-800. The analysis reveals that PNAC-400 possesses a surface charge of −5.54 mV, whereas PNAC-800 displays a significantly more negative surface charge of −15.3 mV. The results imply that PNAC-800 may possess an increased abundance of negatively charged functional groups, such as hydroxyl (–OH) and carboxyl (O=C–O), as corroborated by XPS and FTIR analyses. In summary, PNAC-800 has the highest porosity and surface area, and abundant functionalities, among the series. Therefore, it was selected for all detailed adsorption studies.

### 3.2 Adsorption behaviour of the synthesized adsorbents

The synthesized adsorbents possess diverse surface functional groups, as confirmed by detailed characterizations, enabling effective interactions with contaminants *via* hydrogen bonding, electrostatic attraction, and Lewis acid–base interactions. To identify the optimal adsorbent, the dye adsorption capacities of the PNAC series were evaluated using UV-vis spectroscopy after 20 minutes of equilibrium, and the results are shown in Fig. S4. Among the samples, PNAC-800 exhibited the highest removal efficiencies for all tested dyes. The removal efficiencies are shown in Table S1. Hence, subsequent studies focused exclusively on PNAC-800. All experiments were replicated in triplicate for reproducibility.

**Fig. 4:**
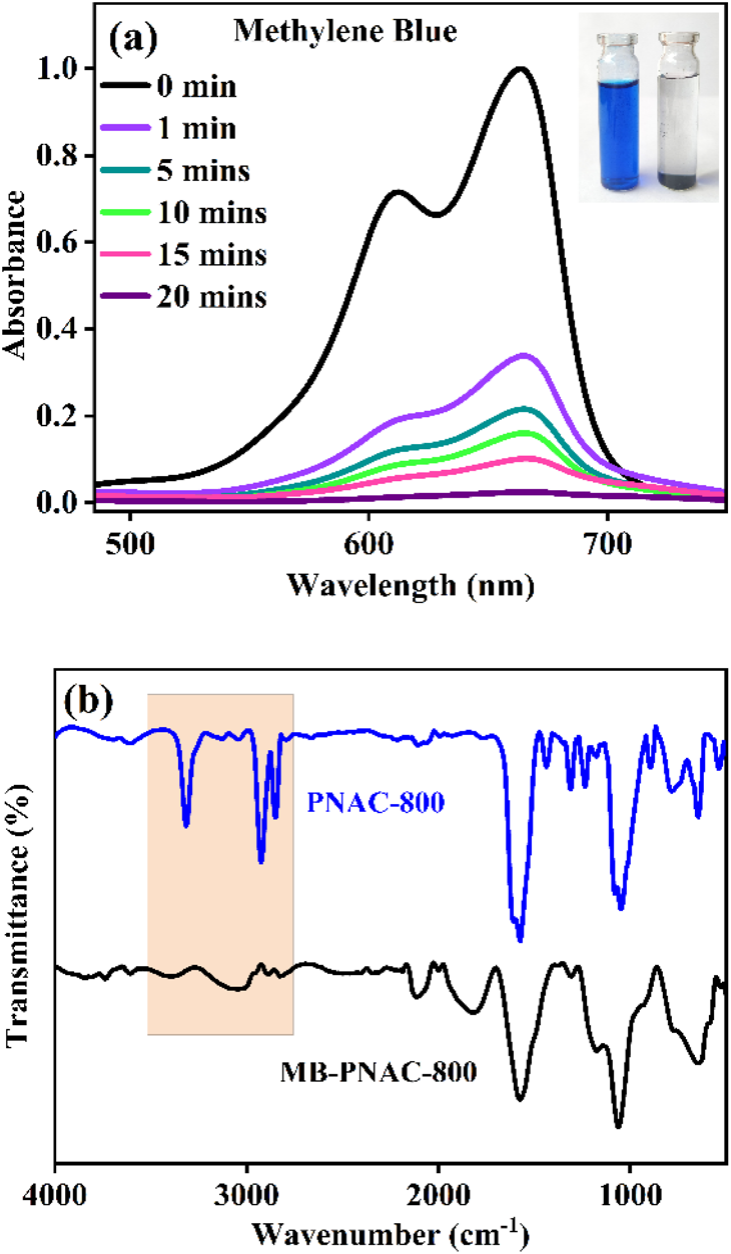
UV-vis spectra of (a) adsorption of MB dye in the presence of adsorbent PNAC-800 (experimental conditions – pH 9, adsorbent dose 0.6 g, initial dye concentration – 80 mg L^−1^ for MB, temperature – 30 °C, contact time – 0 to 20 minutes). (b) The FTIR spectra of PNAC-800 before and after the adsorption of the MB dye.

For MB dye, with a characteristic absorption peak at 664 nm (Fig. 4a), a progressive decline in absorbance intensity confirmed its adsorption onto PNAC-800 within 20 minutes. This adsorption was further validated by FTIR and SEM-EDX analyses. Post-adsorption FTIR spectra (Fig. 4b) reveal surface interactions between PNAC-800 and MB. The broad O–H/N–H stretching band at 3300–3400 cm^−1^ in pristine PNAC-800, arising from hydrogen-bonded hydroxyl and nitrogen-containing groups within the material structure, decreases markedly after MB adsorption. Additionally, aliphatic C–H features near 2900 cm^−1^ show slight attenuation. These spectral changes indicate that both hydroxyl, nitrogen-containing sites, and C–H-bearing moieties participate in MB binding. The pronounced reduction in –OH peak intensity strongly supports the critical role of surface hydroxyl and N-containing functionalities as active adsorption centers for MB. SEM images before and after adsorption (Fig. 2d and S5a) showed that the initially porous surface of PNAC-800 became smoother with diminished pore visibility, indicating dye occupancy. EDX spectra (Fig. 2e and S5b) displayed increased weight and atomic percentages of C, N, and O elements, alongside the appearance of sulphur (S), directly evidencing MB adsorption on the adsorbent surface. Similarly, the adsorption of BG and CV dyes by PNAC-800 was confirmed by UV-vis spectral shifts and complete decolorization within 18 and 14 minutes, respectively (Fig. S6a and c). Corresponding FTIR spectra post-adsorption (Fig. S6b and d) further corroborated the successful dye removal. To substantiate the superior adsorption performance of PNAC-800, its capacities were benchmarked against previously reported adsorbents for cationic dyes, and comparative data are summarized in Table 1.

**Table 1:** Cationic dye adsorption capacities of activated carbons prepared using different biomass and activation agents

S. no.	Carbon adsorbent from various sources	Material used for activation	Surface area (m^2^ g^−1^)	Contact time (min)	Pollutants (dyes)	Adsorption capacity (mg g^−1^)
1	Banana stem (present study)	KOH	1978.8	18–20	MB, BG and CV	1622.99, 1435.97 and 1214.04
2	Peanut shell^47^	FeCl_3_·6H_2_O	722.3	120	Malachite green	747.03
3	Corncobs^48^	KOH and heat	2308.2	360	Methylene blue	523.18
4	Rice husk^49^	KOH	188.57	180	Malachite green	217.6
5	Dex-MA/PAA hydrogel^50^	—	—	5 and 15	Methylene blue and crystal violet	1994 and 2390
6	Activated carbon decorated montmorillonite^51^	H_3_PO_4_	199.64	120	Methylene blue and crystal violet	801.7 and 1110.8
7	Carbonyl-interfaced biochar (almond skin)^52^	—	305.2	10	Methylene blue	3086
8	Activated carbon from Moroccan *Moringa oleifera* wastes^53^	H_3_PO_4_	1394	60	Crystal violet	469.55
9	Indian jujube seeds^54^	H_3_PO_4_	571	60	Acriflavine and victoria blue B	113.6 and 92.78
10	Cotton stalks^55^	KOH	950.0	60	Methylene blue	222
11	Banana stem^56^	H_3_PO_4_	837.4	90	Methylene blue	101.01
12	Pineapple waste biomass^57^	ZnCl_2_	914.6	120	Methylene blue	288.34
13	Cherry tree wood^58^	H_3_PO_4_	738.17	90	Cationic red 14	348.62
14	Mesquite wood chips^59^	CO_2_ and steam	776.4	240	Rhodamine B	189.83
15	Rattan waste^60^	NaOH	1135.0	480	Methylene blue	359

#### 3.2.1 Determination of point zero charge

**Fig. 5:**
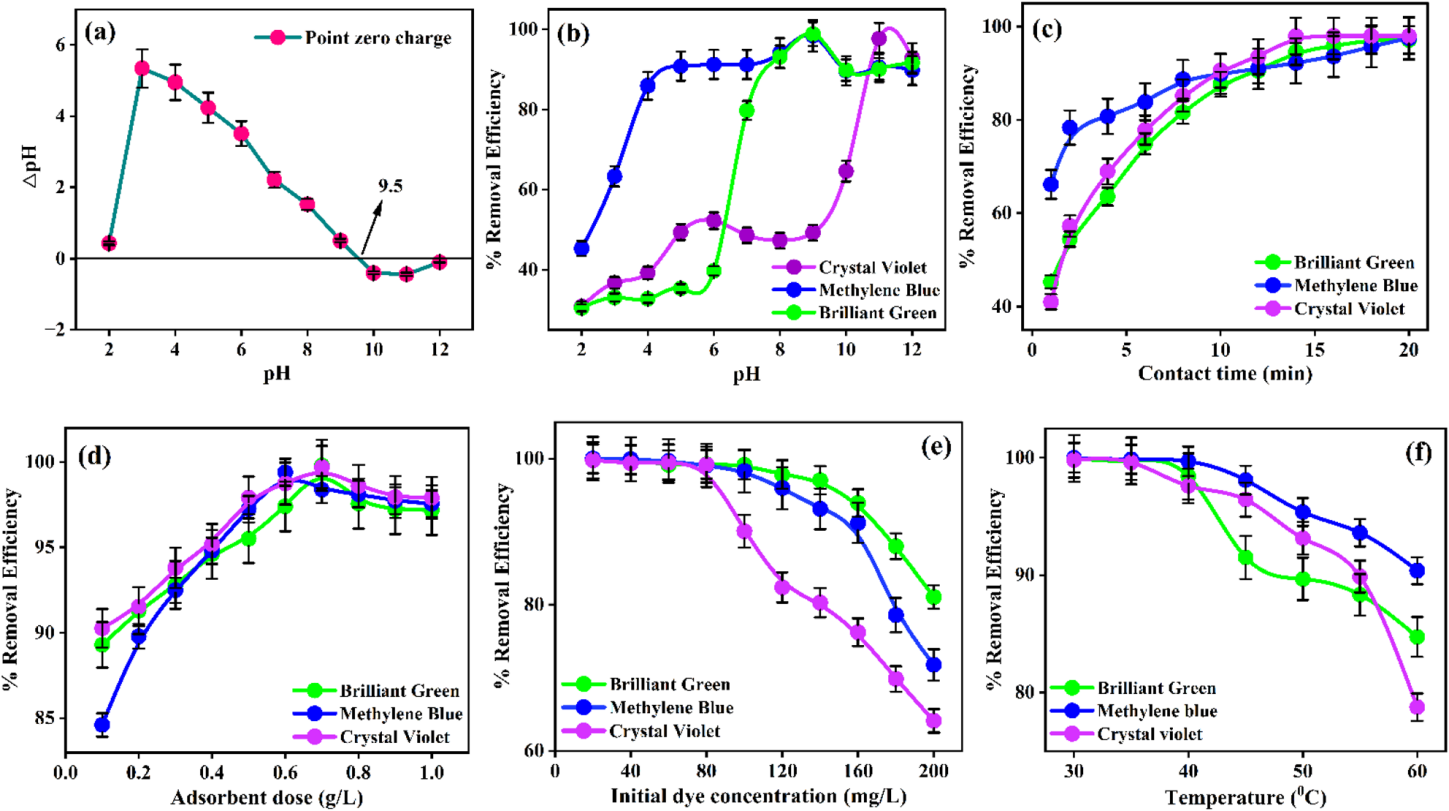
(a) Determination of point zero charge for PNAC-800. The optimization of the adsorption parameters of cationic dyes onto the PNAC-800 adsorbent material (b) effect of pH, (c) effect of contact time, (d) effect of adsorbent dosage, (e) effect of initial dye concentration and (f) effect of temperature.

The point of zero charge (PZC) of the adsorbent was determined using the salt addition method. A series of 100 mL Erlenmeyer flasks was each filled with 45 mL of 0.1 M NaCl solution, which served as a background electrolyte to maintain a constant ionic strength during the experiment. The pH of these solutions was adjusted from 2 to 12 using 0.1 M HCl and 0.1 M NaOH.^61^ Subsequently, 500 mg of PNAC-800 was added to each flask. After 24 hours of equilibration, the final pH values were recorded. The difference between the initial and final pH values (ΔpH) was plotted against the initial pH. The point at which ΔpH equals zero represents the PZC of PNAC-800. As shown in Fig. 5a, the PZC is observed at pH 9.5. At pH values below the PZC (pH < 9.5), the PNAC-800 surface is positively charged, which disfavours the adsorption of cationic dye species due to electrostatic repulsion. Conversely, at pH values above the PZC (pH > 9.5), the surface acquires a negative charge, significantly enhancing the adsorption of cationic dyes through strong electrostatic attraction. The use of NaCl as an inert electrolyte minimizes variations in ionic strength and reduces potential errors associated with pH drift, ensuring more reliable determination of the PZC.

#### 3.2.2 Effect of pH

A study is conducted to examine the adsorption behaviour of the dyes onto PNAC-800, focusing on the effect of pH on the adsorption efficiency. The adsorption performance has been assessed across a pH range of 2 to 12 by comparing the initial and equilibrium concentrations of the dye solutions. The results reveal that pH plays a pivotal role in determining adsorption efficiency.^62,63^ At lower pH levels (2–6), the adsorption capacity is significantly reduced, while increasing the pH to a range of 7–12 led to a marked improvement in dye removal, with maximum adsorption observed at pH 9 for both MB and BG and pH 11 for CV (Fig. 5b). The lower removal efficiency at acidic pH is attributed to the protonation of the PNAC-800 surface, which creates a positive charge on the adsorbent. This results in electrostatic repulsion between the adsorbent and the cationic dye molecules, hindering effective adsorption. As the pH increases, the adsorbent surface becomes negatively charged, enhancing electrostatic attraction between the adsorbent and the dyes, thereby facilitating greater adsorption at alkaline pH. Additionally, non-electrostatic interactions, including van der Waals forces, π–π stacking, and hydrogen bonding, also contributed to the overall adsorption mechanism, further improving dye uptake.

#### 3.2.3 Effect of contact time

The adsorption contact time is a crucial parameter in the dye removal process from wastewater using an adsorbent, as it dictates the time required for the adsorbent to achieve equilibrium interaction with dye molecules, facilitating the transfer of dyes from the aqueous phase to the solid adsorbent. In this study, adsorption of CV, MB, and BG dyes has been observed within one minute, achieving removal efficiencies of 41%, 66.1%, and 45.3%, respectively. The adsorbent, PNAC-800, has a hierarchical pore structure composed of micropores, mesopores, and macropores. The micropores and mesopores are primarily responsible for the initial fast adsorption, followed by a decrease in the adsorption rate as equilibrium approaches. Maximum adsorption capacities are reached at different times for each dye. CV attained equilibrium in 14 minutes, achieving a removal efficiency of 97.9%, while MB and BG reached their maximum adsorption at 20 and 18 minutes, with removal efficiencies of 97.5% and 97.1%, respectively. The removal efficiency of PNAC-800 with different dyes is shown in Fig. 5c. Despite variations in the time to reach equilibrium, the removal efficiencies are consistently high across all dyes, indicating uniform adsorption performance by PNAC-800 (conditions dye concentration – 80 mg L^−1^, adsorbent dose – 0.5 g, pH – 9 for MB and BG, pH – 11 for CV, temperature – 30 °C).

#### 3.2.4 Effect of adsorbent dosage

Determining the optimal adsorbent dosage is critical for maximizing the interaction between adsorbate molecules and available adsorption sites on the adsorbent surface. As illustrated in Fig. 5d, the removal efficiency of the dyes increases with the dosage of PNAC-800, reaching maximum removal efficiencies of 99.7%, 99.8%, and 99.4% at dosages of 0.7, 0.7, and 0.6 g for CV, BG, and MB, respectively. This enhanced removal efficiency can be attributed to the increased availability of active adsorption sites and a greater surface area, facilitating more efficient interactions between the adsorbent and dye molecules. However, beyond these optimal dosages, further increases in the PNAC-800 concentration do not significantly enhance the dye removal efficiency, as evident in Fig. 5d. This plateau effect is likely due to the saturation of adsorbate molecules in the solution, where excess adsorbent remains underutilized due to the lack of available dye molecules for further adsorption.^64,65^ Thus, optimizing the adsorbent dosage is essential to balance maximizing adsorption capacity while avoiding the addition of an unnecessary excess amount of adsorbent, which may result in diminished adsorption efficiency.

#### 3.2.5 Effect of initial dye concentration

To study the effect of initial dye concentration, dye concentrations ranging from 20 to 200 mg L^−1^ were prepared from the stock solution. In these solutions, specific amounts of PNAC-800 are added, especially 0.7 g for BG and CV, and 0.6 g for MB. The removal efficiency of 99.9% is obtained for BG at the initial concentration of 20 mg L^−1^, using 0.7 g of PNAC-800, as shown in Fig. 5e. The removal efficiency of BG is slightly decreased to 99.1% as the dye concentration is raised to 100 mg L^−1^. The removal efficiency of BG, as the concentration of the dye is raised further to 200 mg L^−1^, is progressively reduced to 81%. A similar observation has been realized for MB (Fig. 5e). At a 20 mg per L concentration of MB, the removal efficiency amounted to 100% with 0.6 g of adsorbent. The performance is maintained at a high level until 80 mg L^−1^ of dye concentration and decreases slowly from that concentration to reach 71.7% at a concentration of 200 mg L^−1^. In the case of CV, 99.7% removal is observed at a concentration of 20 mg L^−1^, using 0.7 g of PNAC-800 (Fig. 5e). Even though the removal efficiency remains at around 99% up to 80 mg per L concentration of CV, a further increase in concentration (200 mg L^−1^) progressively decreased efficiency to only 64%. Higher concentrations of the dyes decrease the removal efficiency since high concentrations saturate the available active sites on the surface of the adsorbent. However, at low concentrations, there are usually enough available active sites for the dyes to be well absorbed.^66,67^

#### 3.2.6 Effect of temperature

The experimental results of the effect of temperature on the removal efficiency of the dyes indicate a direct correlation between temperature and the physical bonding of the dye with the active sites of the adsorbent, as shown in Fig. 5f. As the temperature rises, the removal efficiency of the dyes shows a slight decrease. The cationic dyes show around 99.9% removal efficiency using PNAC-800 at 30 °C. However, a continuous increase in temperature significantly impacts dye adsorption, leading to a decrease in adsorption capacity and removal efficiency due to the disruption of intermolecular hydrogen bonding between the dye and the adsorbent in the solution.

#### 3.2.7 Selective adsorption of the dyes

**Fig. 6:**
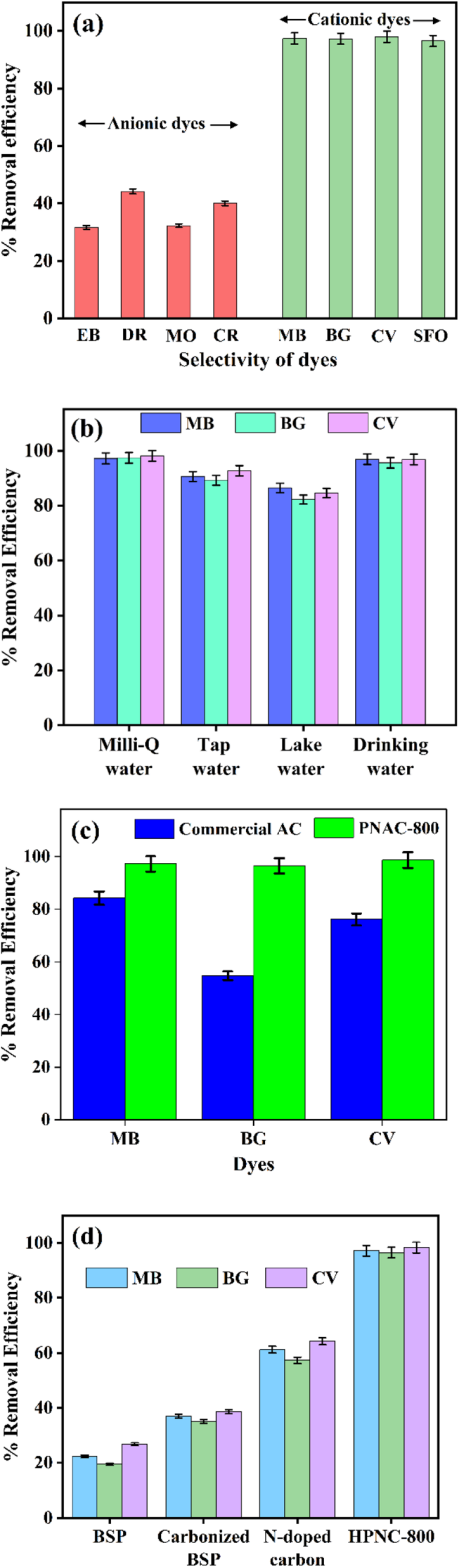
(a) PNAC-800 material selectivity for adsorption, (b) effect of adsorption of cationic dyes from different water sources (experimental conditions – adsorbent dose – 0.7 g, dye concentration – 50 mg L^−1^, neutral pH, temperature – 30 °C, time – 20 min). (c) Comparison of dye removal efficiency between CAC and PNAC-800 for MB, BG, and CV under identical conditions, (d) the adsorption efficiencies of BSP, carbonized BSP, N-doped carbon and PNAC-800 against the cationic dyes (MB, BG and CV dyes).

Fig. 6a illustrates the adsorption capabilities of PNAC-800 for various dyes, highlighting the percentage of removal efficiency for both anionic and cationic dyes. The results indicate a pronounced efficacy in the removal of cationic dyes compared to their anionic counterparts. Notably, MB, BG, CV, and Safranin O (SFO) demonstrate clearance rates exceeding 96%. In stark contrast, the removal efficiencies for anionic dyes such as Eosin B (EB), Direct Red (DR), Methyl Orange (MO), and Congo Red (CR) range between 30% and 50%. This disparity in adsorption efficacy can primarily be attributed to electrostatic interactions. The pronounced removal rates for cationic dyes suggest that the surface of PNAC-800 is predominantly negatively charged at the experimental pH 7. This charge profile facilitates the strong electrostatic attraction of positively charged dye molecules. Conversely, the low adsorption of anionic dyes is a direct consequence of electrostatic repulsion between the negatively charged dye molecules and the anionic surface of PNAC-800. While non-electrostatic interactions (*e.g.*, π–π stacking, van der Waals forces) may still occur, they are insufficient to overcome this primary repulsive barrier. The data underscores PNAC-800's significant potential for the selective removal of cationic dyes from wastewater, as evidenced by the high removal rates of MB and BG, which are approximately 98–99%. This selective adsorption behaviour positions PNAC-800 as a promising candidate for efficient remediation strategies targeting cationic dye pollutants in aqueous environments.

#### 3.2.8 Effect of different water sources on the adsorption efficiency by PNAC-800

The adsorption performance of PNAC-800 for cationic dyes was tested in various water sources. In Milli-Q and drinking water, dye removal efficiencies exceeded 95%, showing excellent performance. In tap and lake water, efficiency slightly dropped but remained above 85%. This decrease is likely due to ions and impurities present in natural water that hinder the interactions between the dye and the adsorbent. Overall, PNAC-800 consistently showed high efficiency across all water types, highlighting its effectiveness in diverse water matrices and its potential for practical wastewater treatment applications. Fig. 6b summarizes the comparative adsorption results.

#### 3.2.9 Comparison of commercial activated carbon and PNAC-800

The adsorption performance of PNAC-800 was benchmarked against commercial activated carbon (CAC) under identical conditions. PNAC-800 consistently achieved higher dye removal efficiencies (MB: ∼95%, BG: ∼96%, CV: ∼97%) compared to CAC (55–85%) (Fig. 6c). Performance evolution from raw banana stem powder (BSP) to PNAC-800 was also studied, and the results are shown in Fig. 6d. The raw BSP showed poor efficiency (∼20–27%) due to its low surface area and amorphous nature. Carbonization improved the porosity of the material, resulting in higher efficiency (∼35–39%) and nitrogen doping further enhanced dye affinity (∼60–65%). Finally, PNAC-800 exhibited near-complete removal (∼98%), attributed to its hierarchical porosity, high surface area, and nitrogen functionalities.

### 3.3 Adsorption isotherms

Adsorption isotherms provide essential insights into the equilibrium distribution of solutes at the solid–liquid interface and are key for understanding adsorption mechanisms and surface interactions.^68,69^ In this study, equilibrium data for cationic dyes on PNAC-800 were analyzed using the Langmuir and the Freundlich models, which are widely applied for evaluating adsorption processes and surface characteristics.

**Fig. 7:**
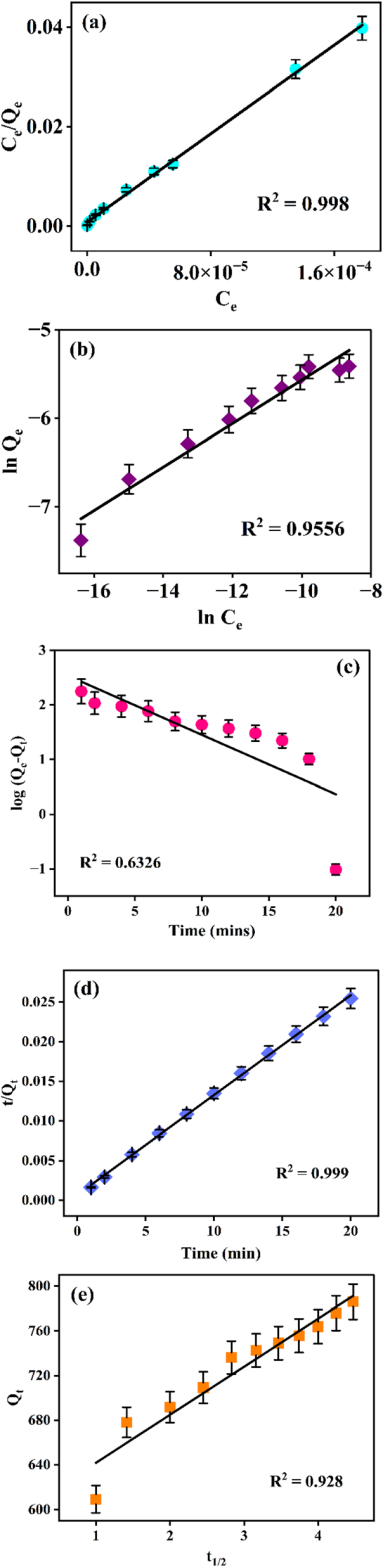
Graphical illustrations of adsorption isotherm models of MB on PNAC-800: (a) Langmuir isotherm fit, (b) Freundlich isotherm fit. Graphical representations of adsorption kinetics of methylene blue on PNAC-800: (c) pseudo-first-order model fit, (d) pseudo-second-order model fit, and (e) intraparticle diffusion model fit.

The Langmuir model exhibited superior correlation coefficients for all studied dyes (*R*^2^ ≈ 1) in Fig. 7a and S7(a), (c), compared to the Freundlich model in Fig. 7b and S7(b), (d) it signifying that dye adsorption occurred primarily through monolayer coverage on a homogeneous surface with uniform active sites. The derived Langmuir parameters demonstrated high monolayer adsorption capacities given in Table 2, exceeding those of several reported adsorbents, indicating the excellent affinity and efficient binding of PNAC-800 toward cationic dyes. The dimensionless separation factor (RL) values obtained for all dyes were between 0 and 1, confirming the favourable and spontaneous nature of the adsorption process.

**Table 2:** Adsorption isotherm parameters of the Langmuir and the Freundlich

Isotherm model	Parameters	Methylene blue	Brilliant green	Crystal violet
Langmuir	*Q*_max_ (mg g^−1^)	1435.97	1622.99	1214.04
	*K*_L_ (L g^−1^)	0.87	0.88	0.27
	*R* ^2^	0.9980	0.9980	0.9593
Freundlich	1/*n*	0.2458	0.2483	0.2165
	*K*_f_ (mg g^−1^)	248.96	276.88	46.25
	*R* ^2^	0.9556	0.9689	0.8387

Although the Freundlich model also showed reasonable linearity (*R*^2^ > 0.8387 for CV), it provided a comparatively weaker fit, revealing minor contributions of multilayer adsorption, particularly for brilliant green (0.9689) and methylene blue (0.9556). The calculated 1/*n* values (0.1–1) further confirmed effective adsorption and moderate surface heterogeneity. Collectively, these results indicate that the adsorption of cationic dyes onto PNAC-800 is mainly governed by monolayer chemisorption on uniform surface sites, with limited multilayer interactions.

### 3.4 Adsorption kinetics

The adsorption kinetics of MB, BG, and CV dyes onto NPAC-800, synthesized by KOH activation of banana stems, were analyzed using several kinetic models. These include the pseudo-first-order, pseudo-second-order, and intraparticle diffusion models, as shown in the provided plots. The rate constants and linear regression coefficient values of pseudo-first, second and intraparticle diffusion models are given in Table S2.

The pseudo-first-order kinetic plots for all dyes (MB, BG and CV) show relatively low correlation coefficients (*R*^2^ = 0.6326, 0.6699, and 0.7328, respectively, Fig. 7c and S8a, d), indicating poor fit and suggesting that the adsorption process does not primarily follow physisorption-related first-order kinetics. In contrast, the pseudo-second-order model exhibits an excellent fit to the experimental data for all three dyes, evidenced by the high *R*^2^ values 0.999, 0.9979, and 0.9988 in linear fits, Fig. 7d and S8b, e. This strong linear correlation signifies that the adsorption rate is likely controlled by chemisorption between the dye molecules and the functional groups on NPAC-800. The intraparticle diffusion plots *Q*_*t*_*versus t*^1/2^ display multilinearity (*R*^2^ = 0.928, 0.9582 and 0.9151, respectively, with linear fits in Fig. 7e and S8c, f) for each dye, which suggests that the adsorption mechanism involves more than one rate-controlling step. The initial sharper region corresponds to surface adsorption or boundary layer diffusion, followed by a second, gradual linear phase attributed to intraparticle diffusion. The kinetic model in this study highlights multiple rate-controlling steps and adheres to both the pseudo-second order and intraparticle diffusion models.

### 3.5 Thermodynamic parameters of adsorption

Thermodynamic parameters, Gibbs free energy (Δ*G*°), enthalpy (Δ*H*°), and entropy (Δ*S*°) were calculated to elucidate the adsorption mechanism of MB, BG, and CV dyes onto PNAC-800 using the following equations:

3 Δ*G* = −*RT* ln *K*_c_

4
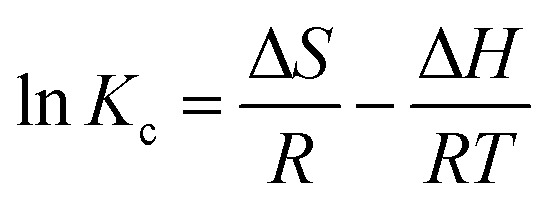
where *K*_c_ is the thermodynamic equilibrium constant, *R* is the universal gas constant, and *T* is the absolute temperature. Van't Hoff plots of ln *K*_c_*versus* 1/*T* (Fig. S9) provided Δ*H*° and Δ*S*°, with values summarized in Table S3. Negative Δ*G*° values confirm that dye adsorption is spontaneous and thermodynamically favourable. Positive Δ*H*° values (162.68, 215.66, and 133.85 kJ mol^−1^ for BG, MB, and CV, respectively) indicate endothermic adsorption. Correspondingly, positive Δ*S*° values (0.48, 0.64, and 0.39 kJ mol^−1^ K^−1^) reflect increased randomness at the solid-solution interface during adsorption.

### 3.6 Reusability and recyclability of PNAC-800

**Fig. 8:**
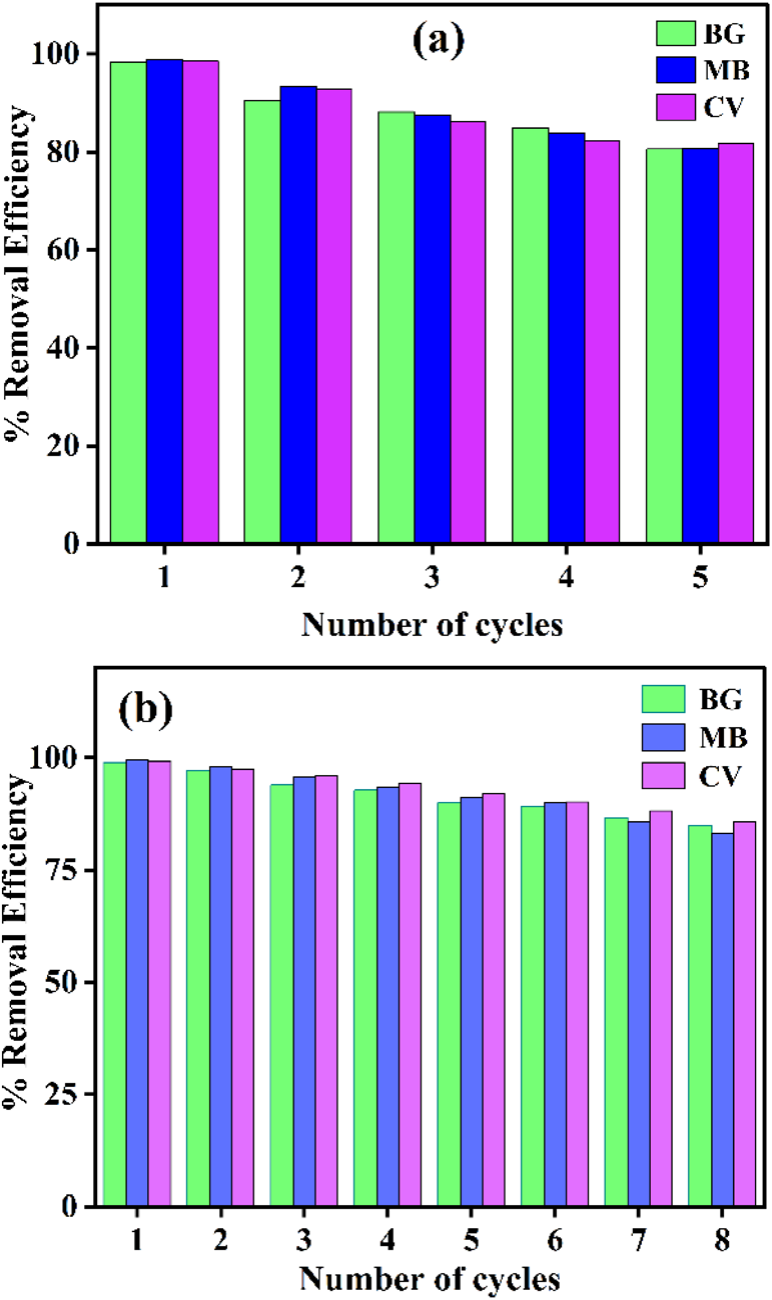
(a) Assessing the reusability of PNAC-800 for the adsorption of BG, MB, and CV without employing a desorption agent. (b) Evaluating the recyclability of PNAC-800 in the adsorption processes of BG, MB, and CV dyes using a desorption agent.

Assessing adsorbent reusability is vital for its practical applicability and cost-effectiveness.^70^ PNAC-800 exhibited an initial dye removal efficiency of ∼80% after five adsorption-drying cycles, with efficiency declining progressively (Fig. 8a). To enhance regeneration, 0.1 M HCl was employed as a desorption agent; after 3 h treatment followed by washing and drying, the adsorbent performance remained stable with negligible efficiency loss up to five cycles and retained 85% efficiency after eight cycles (Fig. 8b). These findings demonstrate that PNAC-800 maintains robust adsorption capacity for BG, MB, and CV dyes across multiple cycles, sustaining performance for five cycles without desorption and up to eight cycles with acid-assisted regeneration, highlighting its excellent reusability and structural stability.

### 3.7 Preliminary test with coloured industrial wastewater

To gain an initial observation of the material's behaviour in a complex matrix, a test was performed using raw, coloured wastewater collected from a local textile facility. When PNAC-800 was introduced to the untreated effluent (visually pink and black samples), rapid decolorization occurred, with approximately 97% reduction in intensity within 15 minutes (Fig. S10a and b). It is important to note that the specific dye classes and auxiliary chemicals present in this uncharacterized effluent were not identified. Therefore, this test serves as an initial observation of the material's decolorizing capability in a complex matrix, rather than as a quantitative validation for specific dye pollutants. These results indicate a promising direction for future research, which requires application to well-defined industrial effluents with known compositions to assess practical performance and potential interference from dyebath additives fully.

### 3.8 Additional adsorption analysis of pharmaceutical drugs using PNAC-800 adsorbent

To extend its applicability beyond dyes, the adsorption performance of PNAC-800 was evaluated against pharmaceutical contaminants using ciprofloxacin (CPX) and cefixime (CFX) as model pollutants. These antibiotics, commonly released into aquatic environments *via* hospital, agricultural, and pharmaceutical effluents, pose significant ecotoxicological risks to algae, microbes, and aquatic larvae.^71–74^ Adsorption experiments were conducted at an initial concentration of 20 mg L^−1^ (0.1 g PNAC-800, 30 minutes contact time, neutral pH), yielding removal efficiencies of 79.8% for CPX and 81.1% for CFX (Fig. S11a and b). These results highlight the potential of PNAC-800 as a promising adsorbent for pharmaceutical remediation, with future studies directed toward optimizing adsorption parameters and assessing performance in real effluents.

### 3.9 Adsorption mechanism of the dyes onto PNAC-800

The adsorption mechanisms of MB, BG, and CV onto PNAC-800 are expected to involve a variety of intricate interactions. These include surface functionalization, multiple adsorption sites, electrostatic attractions, π–π stacking, hydrogen bonding, pore diffusion, and physical adsorption, all of which are influenced by pH dynamics. Our synthesized PNAC-800 exhibits a high surface area, as corroborated by BET, SEM, and TEM analyses, which confirm the hierarchical porous architecture of PNAC-800. The abundance of functional groups on the adsorbent surface is verified through FTIR and XPS studies. The removal efficiency for the three dyes reached more than 97% within 20 minutes of adsorption. Initial rapid adsorption is characterized by a swift 40% removal within the first minute, predominantly driven by pore filling from a boundary layer of the adsorbent for low-concentration organic molecules.^75^ The surface functional groups play an essential role in this adsorption process. Nitrogen doping introduces a variety of nitrogen-containing functional groups, including pyridinic-N, pyrrolic-N, and graphitic-N, thereby enhancing the surface chemistry of adsorption. This modification increases surface polarity and provides Lewis acid–base interaction sites, facilitating electrostatic attractions with the cationic dyes.^30^

Additionally, in the optimal pH conditions (typically around pH 10 and 11), the PNAC-800 surface acquires a negative charge due to the deprotonation of hydroxyl groups, fostering robust electrostatic interactions with the positively charged dye molecules. Conversely, lower pH levels lead to competition between protons and cationic dyes for adsorption sites, diminishing removal efficiency. Beyond electrostatic interactions, the partial graphitic structure of the activated carbon promotes π–π interactions with the organic dyes, enhancing overall adsorption capacity.^75^ The lone pair electrons of the oxygen functional groups may also engage in n–π interactions with the dyes. Furthermore, nitrogen and oxygen functionalities facilitate hydrogen bonding with dye molecules, and the amino groups in the dyes can interact with hydroxyl groups present on PNAC-800. Contributions from pyrrolic-N and pyridinic-N to hydrogen bonding further enhance the adsorption efficiency.

**Fig. 9:**
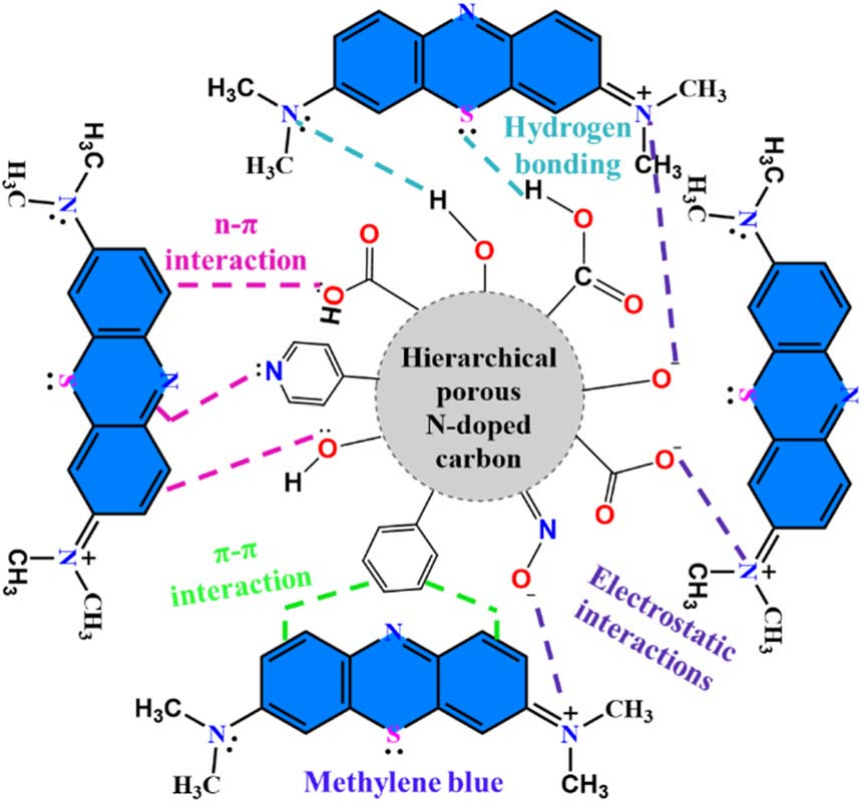
Plausible adsorption mechanism of MB onto the surface of PNAC-800.

In summary, the optimized N-doped carbon sample enhances physical adsorption *via* pore filling, while nitrogen doping also facilitates adsorption through hydrogen bonding, electrostatic interactions, effectively removing dye molecules from the solution. The possible adsorption mechanism of MB dye onto the surface of PNAC-800 is shown in Fig. 9. The other two plausible adsorption mechanisms of BG and CV are illustrated in Fig. S12 and S13, respectively. Additionally, the anticipated model for the adsorption mechanisms of various dyes and pharmaceutical compounds based on pore filling is presented in Fig. S14.

## 4. Conclusions

This study demonstrates the rational design of nitrogen-doped porous activated carbon (PNAC-800) derived from banana plant stems, presenting a sustainable and efficient adsorbent for the removal of cationic dyes and pharmaceutical contaminants. PNAC-800 exhibits an exceptionally high surface area (1978.8 m^2^ g^−1^) and well-engineered surface functionalities that enable outstanding adsorption capacity and rapid kinetics, achieving up to 97% removal of cationic dyes within 20 minutes. The adsorption process conforms to Langmuir isotherm behavior, indicating monolayer coverage, and follows a hybrid pseudo-second-order and intraparticle diffusion kinetic model. Thermodynamic evaluations reveal that the process is spontaneous, endothermic, and energetically favorable, confirming strong interactions between adsorbate molecules and the PNAC-800 surface. Moreover, PNAC-800 retains 80% of its adsorption efficiency even after eight acid-assisted regeneration cycles. It's high performance is demonstrated for synthetic cationic dyes and pharmaceutical pollutant removal and its functionality is shown in a preliminary test with real colored industrial wastewater. The enhanced affinity toward cationic dyes arises from the presence of anionic surface functionalities, emphasizing the tunability of PNAC-800's surface chemistry for selective pollutant capture. The nitrogen-doped porous carbon exhibits low adsorption efficiency toward anionic dyes, which is attributed to electrostatic repulsion between the negatively charged surface and anionic dye molecules. This result highlights the inherent selectivity of the present material toward cationic dyes. Future work will focus on surface modification strategies like tuning surface charge or introducing positively charged functional groups to broaden the applicability of the sorbent toward both cationic and anionic dyes. Overall, this work establishes PNAC-800 as a scalable, bio-derived, and high-performance adsorbent for selectively cationic species, it offering a promising pathway toward sustainable and efficient water purification technologies.

## Author contributions

Alibasha Akbar: led the initial draft, methodology, review, and final editing. M. Bhavani Lakshmi: focused on review and editing for clarity. Tanmay Chatterjee, Paramita Pattanayak, Quazi Arif Islam, and Sritama Mukherjee: handled formal analysis, writing, and technical review. Mihir Ghosh: provided oversight, conceptual framework, and contributed to writing, review, and editing.

## Conflicts of interest

The authors affirm that there are no disclosed financial conflicts of interest or personal affiliations that might be construed as influencing the findings presented in this manuscript.

## Supplementary Material

RA-016-D5RA09071G-s001

## Data Availability

The findings of this study are supported by data that can be obtained from the corresponding authors upon reasonable request. Supplementary information (SI): additional material characterization data (Raman, XPS, zeta potential, SEM-EDX, FTIR), UV-vis spectra for dye and pharmaceutical adsorption studies, removal efficiency tables, kinetic, isotherm and thermodynamic analyses, treatment of real industrial effluents, and proposed adsorption mechanism illustrations. See DOI: https://doi.org/10.1039/d5ra09071g.
