# Supplementary material for: Valorisation of banana stem into N-doped activated carbon as a selective sorbent for cationic dyes and pharmaceutical contaminants
Source: RSC Adv. 2026 Feb 27;16(13):11426–41. doi: 10.1039/d5ra09071g (PMC12947633; doi:10.1039/d5ra09071g)
Supplement: RA-016-D5RA09071G-s001 [file RA-016-D5RA09071G-s001.pdf]

Supplementary Information (SI) for RSC Advances.
This journal is © The Royal Society of Chemistry 2026

Supplementary Information

Valorisation of banana stem into N-doped activated carbon as a selective sorbent for cationic dyes and pharmaceutical contaminants

Alibasha Akbar [a], M. Bhavani Lakshmi [a], Priyadip Das [a], Quazi Arif Islam [b], Tanmay Chatterjee [c], Paramita Pattanayak [c], Sritama Mukherjee [d], Mihir Ghosh [a*]

[a] Department of Chemistry, SRM Institute of Science and Technology, Kattankulathur (SRMIST-KTR), Tamil Nadu 603203, India.

[b] Department of Chemistry, Alipurduar University, P.O.– Alipurduar Court, District- Alipurduar, West Bengal, 736122, India

[c] Department of Chemistry, Birla Institute of Technology and Science, Pilani (BITS Pilani), Hyderabad Campus, Jawahar Nagar, Kapra Mandal, Hyderabad 500078, India.

[d] Division of Fiber and Polymer Technology, CBH, KTH Royal Institute of Technology, Teknikringen 56-58, SE-100 44 Stockholm, Sweden.

1. Figure S1. (a) FITR spectrum of BS hydrochar, (b) Thermogravimetric analysis of BS hydrochar, (c) Raman spectra of PNAC-400, PNAC-550, PNAC-700, and PNAC-800 adsorbents.

2. Figure S2. (a) XPS survey spectra of PNAC-400 (b) C1s core level spectra of PNAC-400, (c) N1s core level spectra of PNAC-400, and (d) O1s core level spectra of PNAC-400.

3. Figure S3. Zeta potential analysis of (a) PNAC-400 and (b) PNAC-800.

4. Figure S4. UV-vis spectra of (a) adsorption of MB dye, (b) adsorption of BG dye, and (c) adsorption of CV dye after 20 minutes of reaching equilibrium in the presence of adsorbents, PNAC-400, PNAC-550, PNAC-700, and PNAC-800.

5. Figure S5. SEM image and EDX spectra of PNAC-800 after adsorption of MB dye.

6. Table S1. Removal efficiencies of MB. BG and CV dyes with synthesized PNAC materials.

7. Figure S6. UV-vis spectra of (a) BG adsorption and (c) CV dye adsorption in the presence of adsorbent PNAC-800. FTIR spectra of before and after adsorption of cationic dyes (b) BG and (d) CV using PNAC-800 adsorbent.

8. Figure S7. Graphical illustration of adsorption isotherm models of BG and CV dyes.

9. Figure S8. Graphical representations of kinetic model fitting plots for the BG and CV dyes onto PNAC-800 adsorbent.

10. Table S2. Adsorption kinetic model parameters of pseudo-first order, pseudo-second order, intraparticle diffusion model, and liquid film model.

11. Figure S9. Thermodynamic parameters from Vant Hoff's plots for MB, MG, and CV dyes

12. Table S3. Thermodynamic parameter values of Gibbs free energy, enthalpy, and entropy.

13. Figure S10. (a) UV-vis spectra depicting the adsorption behaviour of black industrial dye effluent in the presence of the synthesized adsorbent, PNAC-800, and (b) UV-vis spectra illustrating the adsorption characteristics of pink industrial dye effluent when treated with the synthesized adsorbent, PNAC-800.

14. Figure S11. UV-vis spectra illustrating adsorption for (a) Ciprofloxacin and (b) Cefixime using PNAC-800 adsorbent.

15. Figure S12. Plausible adsorption mechanism of BG onto the surface of PNAC-800.

16. Figure S13. Plausible adsorption mechanism of CV onto the surface of PNAC-800 adsorbent.

17. Figure S14. Plausible way of adsorption of dyes (BG, MB, and CV) and pharmaceutical drugs (CPX and CFX) onto PNAC-800 sample by pore filling process.

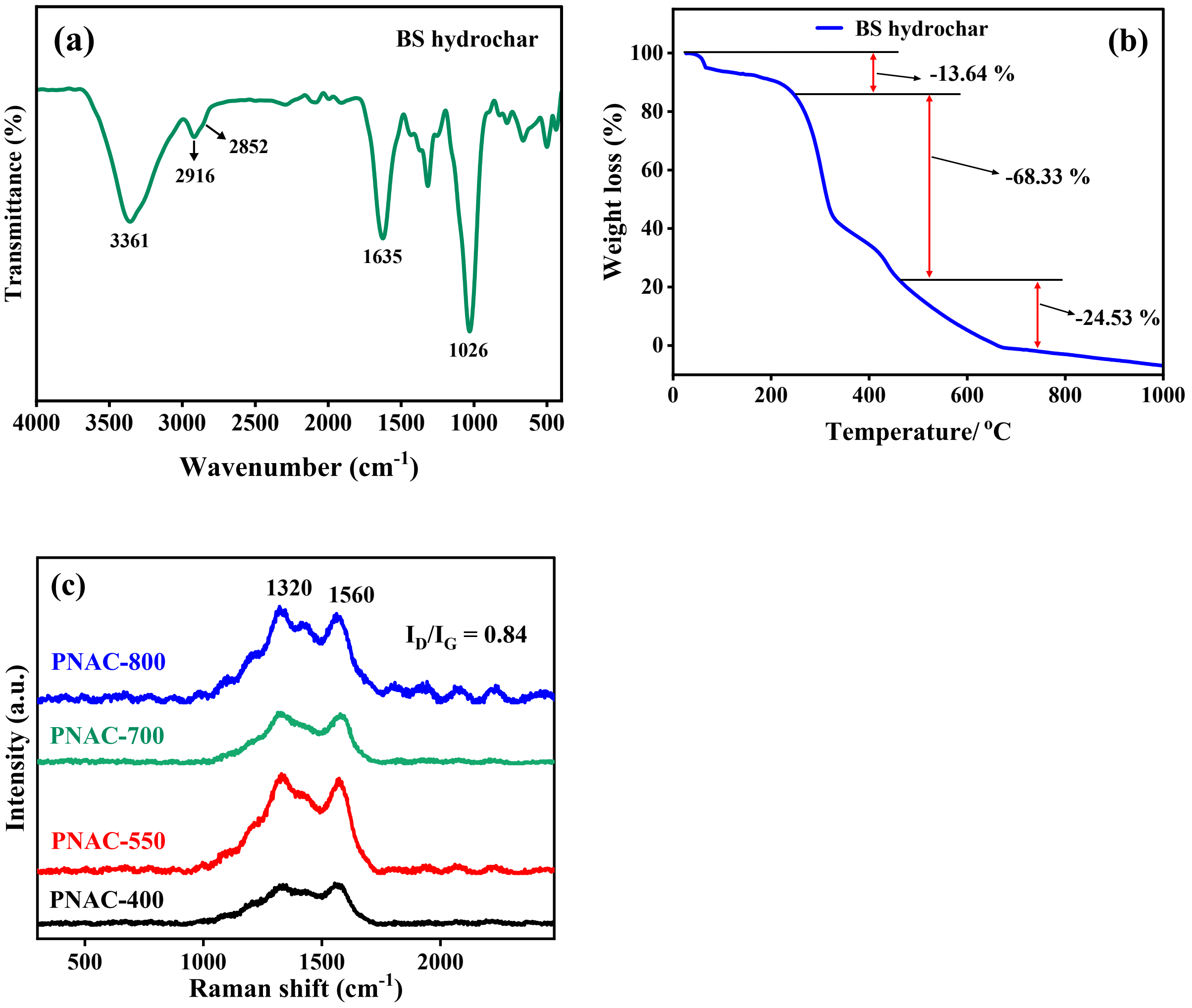

Figure S1. (a) The FTIR spectrum of BS hydrochar after hydrothermal treatment of BS powder (b) Thermogravimetric analysis of BS hydrochar (c) Raman spectra of PNAC-400, PNAC-550, PNAC-700, and PNAC-800 adsorbent materials.

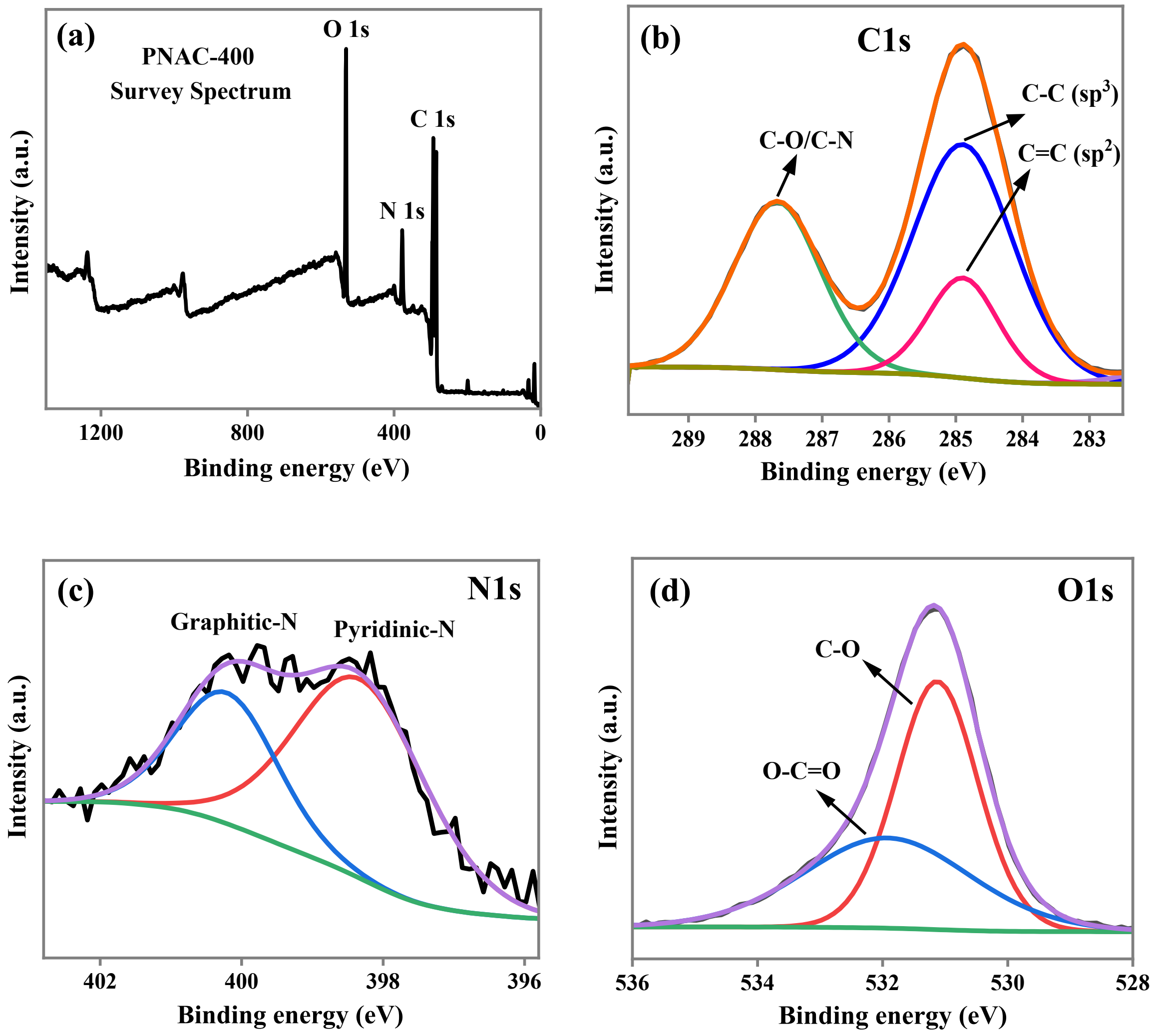

Figure S2. XPS spectra of PNAC-400: (a) Survey spectra, (b) C1s core level spectra, (c) N1s core level spectra, and (d) O1s core level spectra.

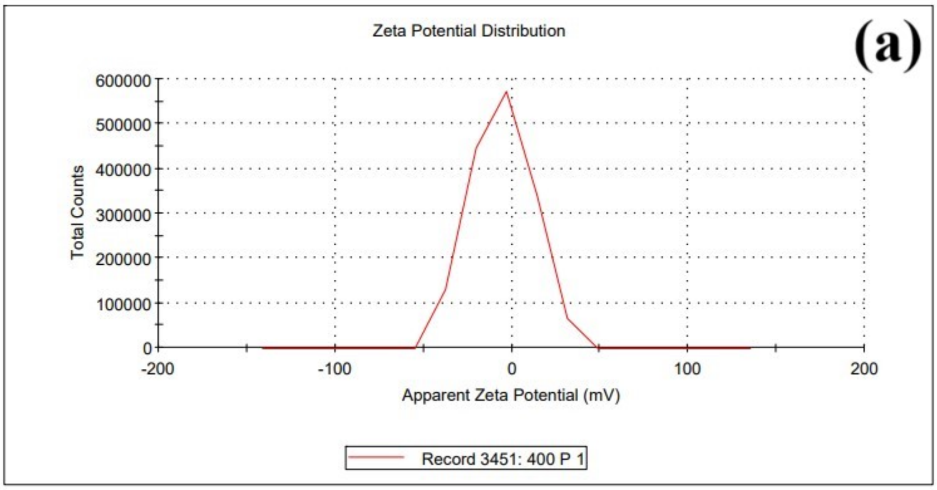

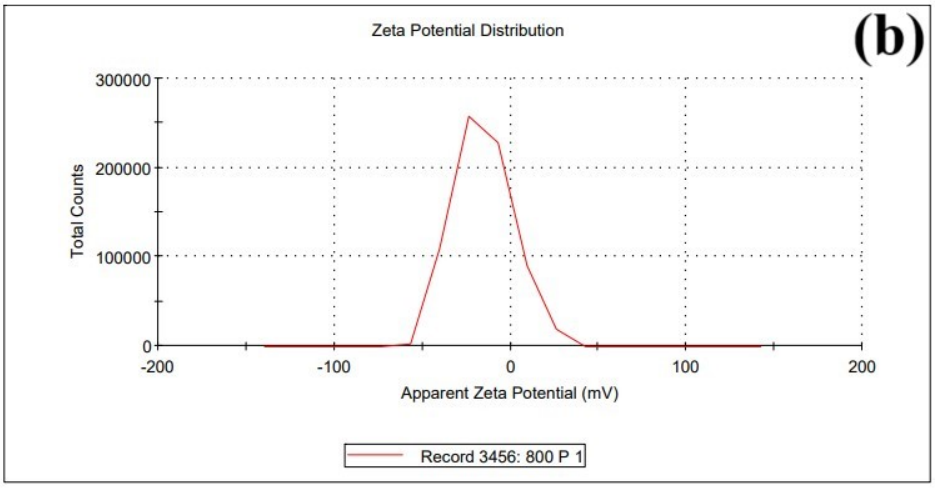

Figure S3. Zeta potential analysis of (a) PNAC-400 and (b) PNAC-800.

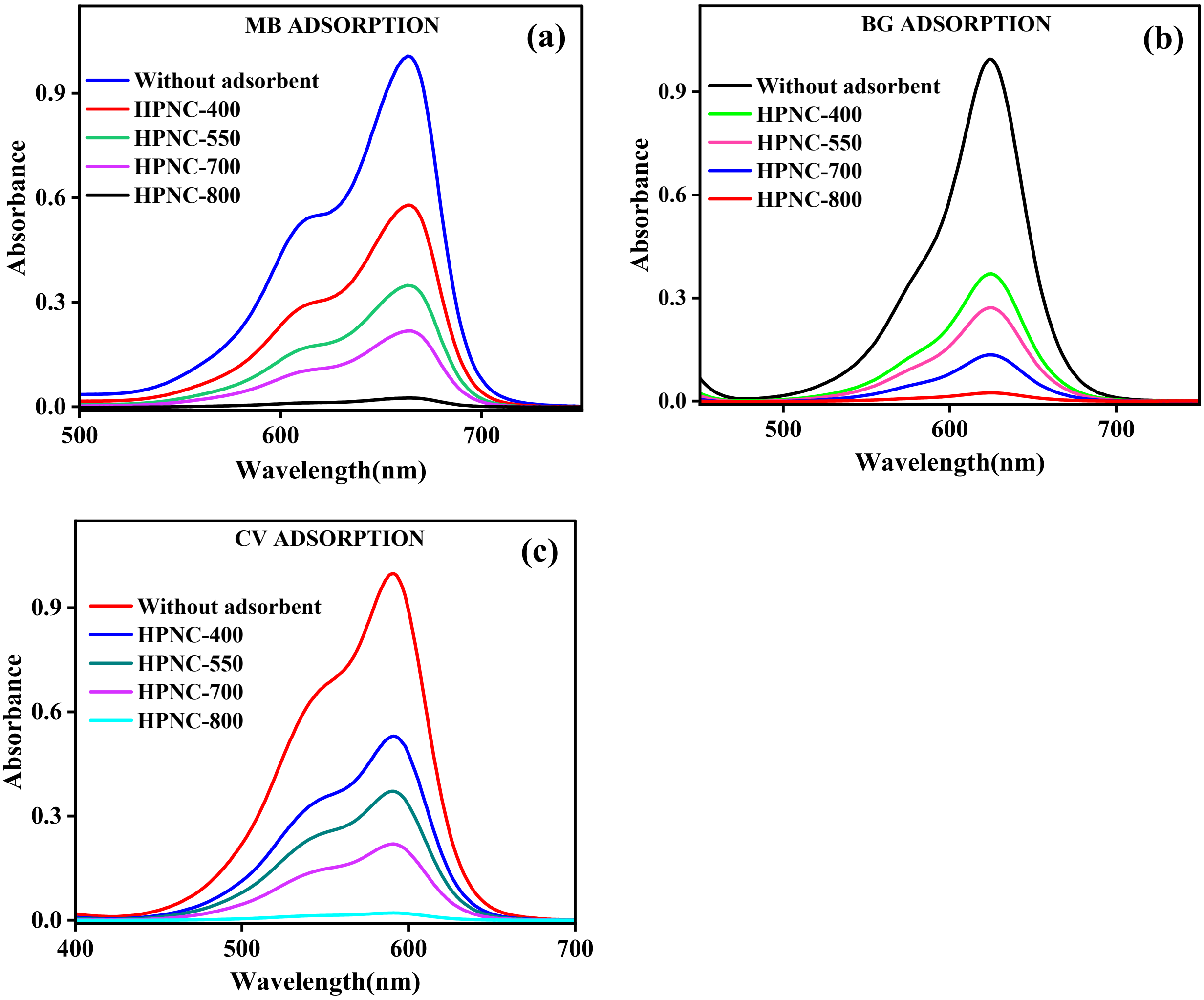

Figure S4. UV-vis spectra of (a) adsorption of MB dye, (b) adsorption of BG dye, and (c) adsorption of CV dye after 20 minutes of reaching equilibrium in the presence of adsorbents, PNAC-400, PNAC-550, PNAC-700, and PNAC-800.

Table S1: Removal efficiencies of MB. BG and CV dyes with synthesized PNAC materials.

**Materials**	**Removal efficiencies of dyes**		
	**Methylene blue**	**Brilliant green**	**Crystal violet**
**PNAC-400**	42.5%	62.8%	46.9%
**PNAC-550**	65.4%	72.7%	62.8%
**PNAC-700**	78.3%	86.4%	78%
**PNAC-800**	99.4%	99.8%	99.7%

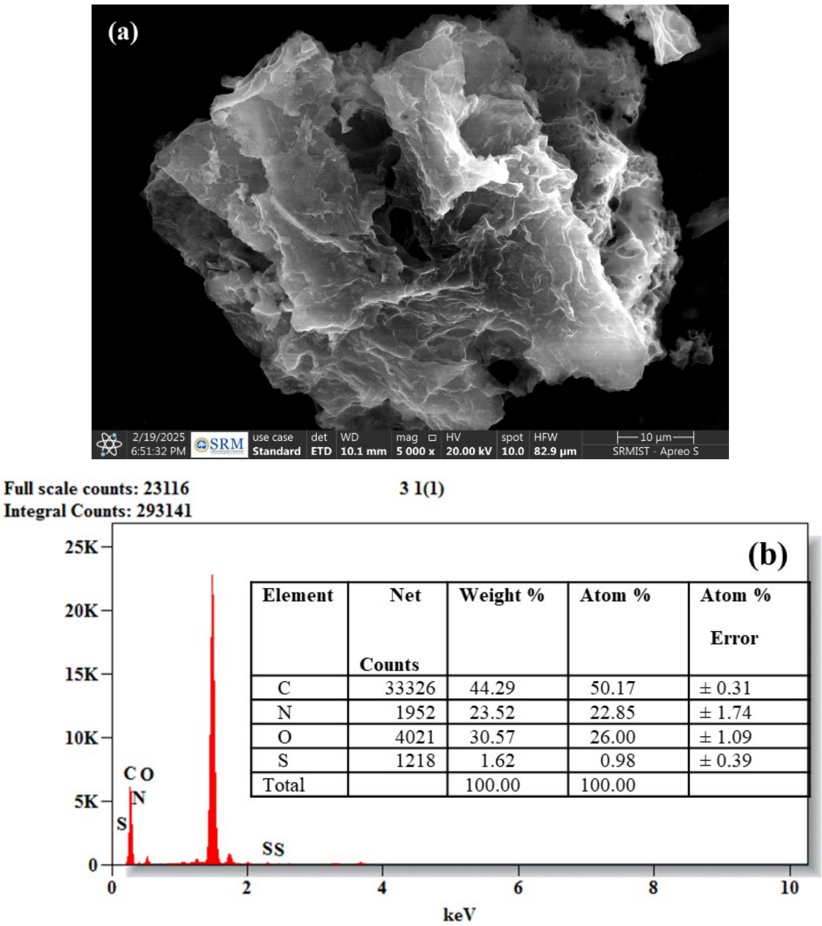

Figure S5. (a) SEM image of PNAC-800 after adsorption of MB dye and (b) corresponding EDX spectra.

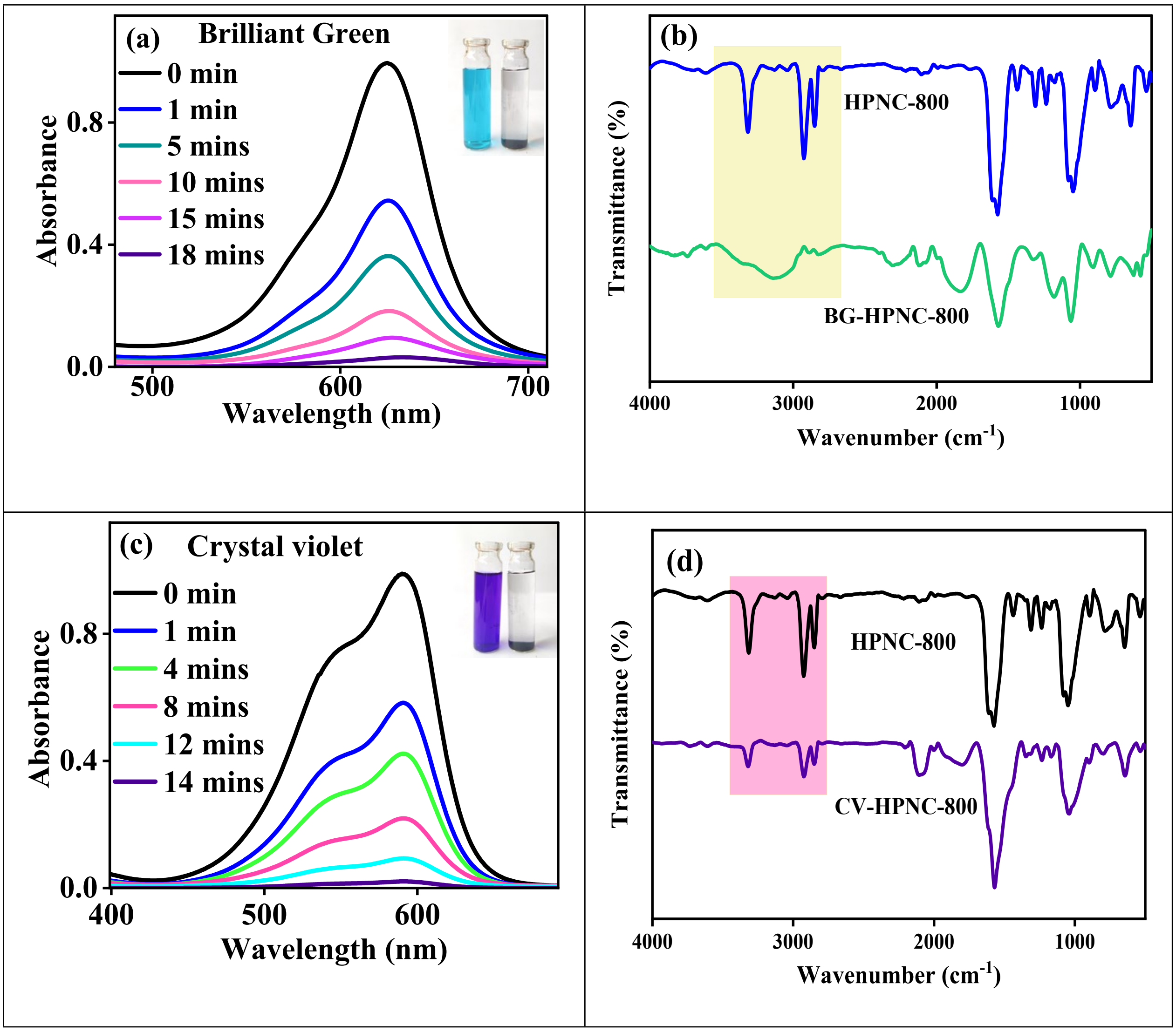

Figure S6. UV-vis spectra of (a) adsorption of BG dye and (c) adsorption of CV dye in the presence of adsorbent PNAC-800. FTIR spectra of before and after adsorption of cationic dyes (b) BG and (d) CV using PNAC-800 adsorbent.

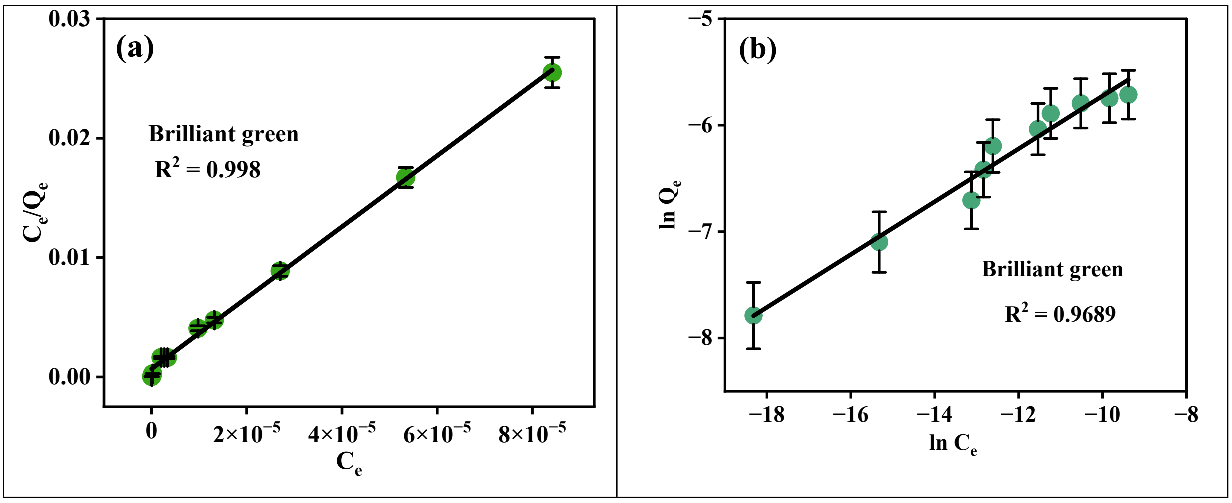

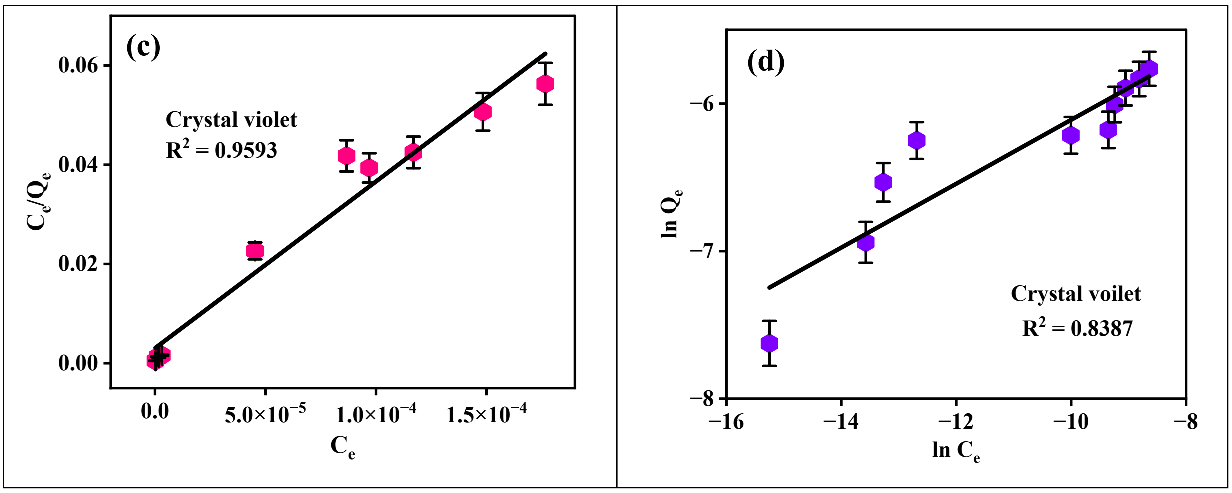

Figure S7. Graphical illustration of brilliant green and crystal violet adsorption isotherm models (a) BG Langmuir fit, (b) BG Freundlich isotherm fit, (c) CV Langmuir fit, (d) CV Freundlich isotherm fit.

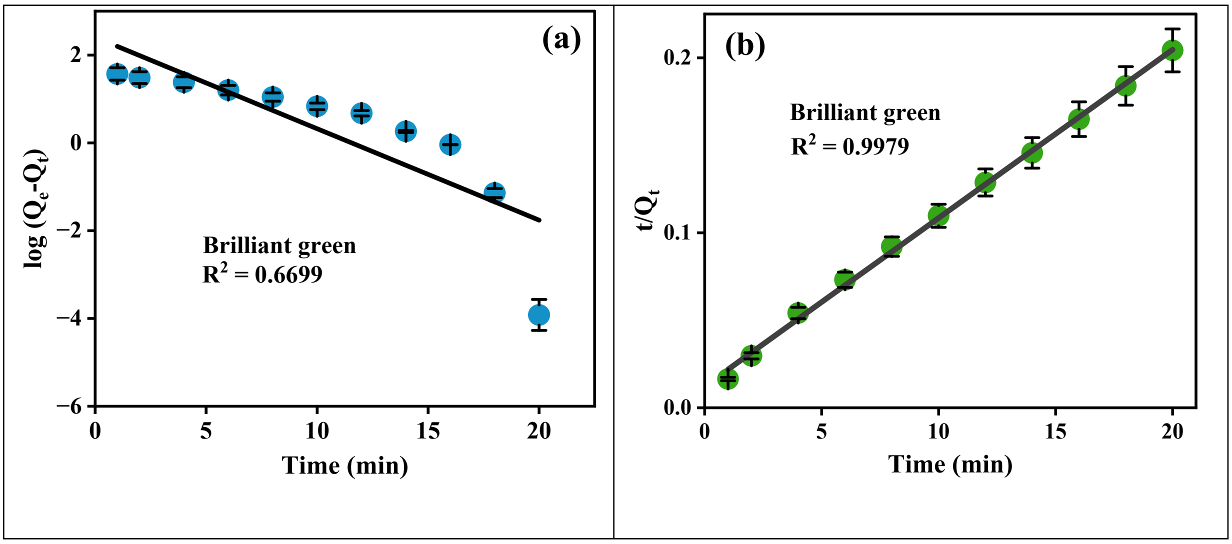

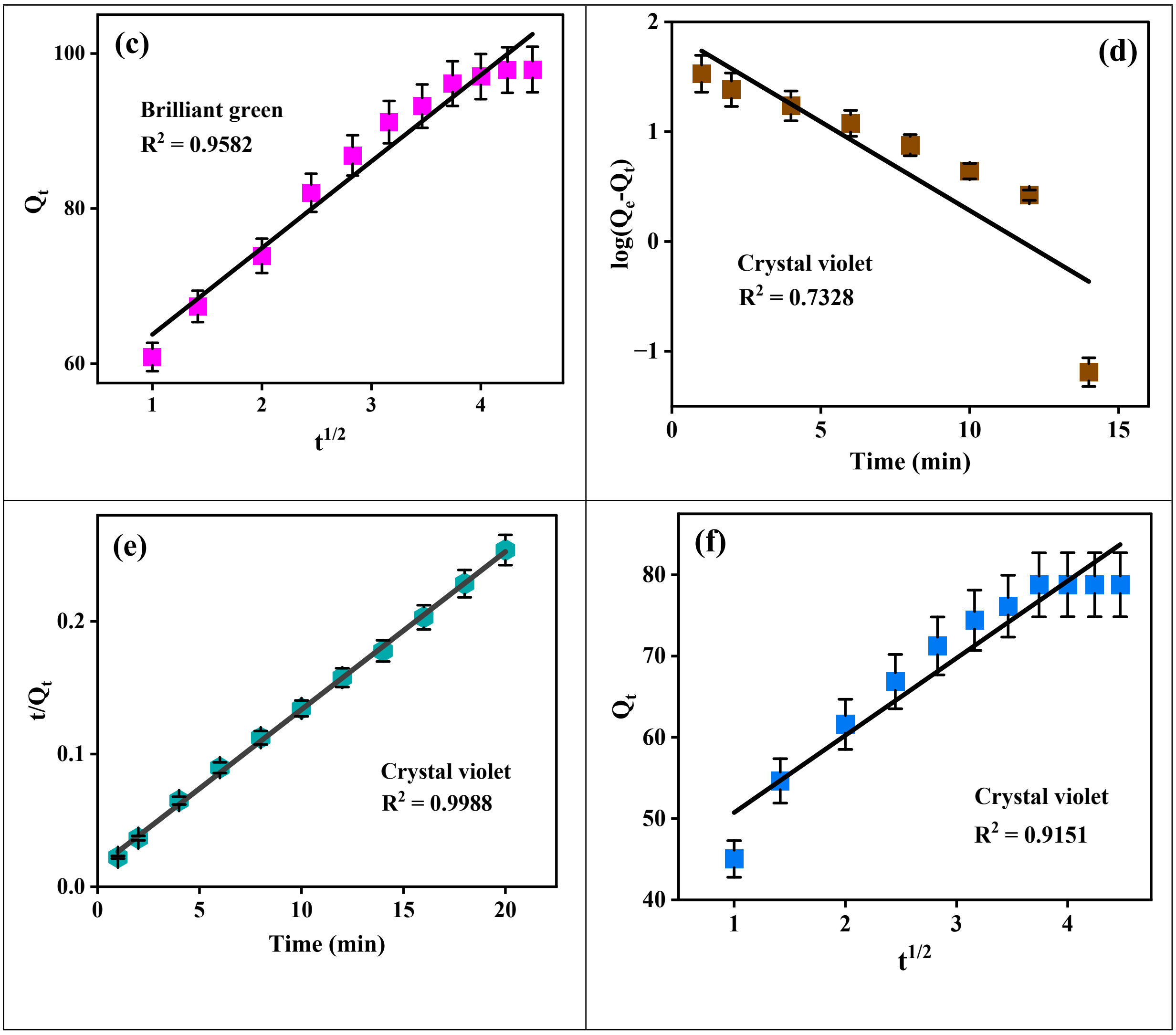

Figure S8. Kinetic fitting plots for the brilliant green and crystal violet dyes onto PNAC-800 (a) and (d) pseudo-first-order for BG and CV dyes. (b) and (e) pseudo-second-order for BG and CV dyes. (c) and (f) Weber-Morris intraparticle diffusion kinetic model.

Table S2. Adsorption kinetic model parameters of pseudo-first order, pseudo-second order, intraparticle diffusion model, and liquid film model.

**Kinetic model**	**Parameters**	**Methylene blue**	**Brilliant green**	**Crystal violet**
	K_1 (min^{-1})	0.25	0.47	0.37
**Pseudo-first order**	Q_e – cal (mg/g)	34.42	254.80	79.07
	Q_e – exp (mg/g)	78.61	97.94	78.78
	R^2	0.6326	0.6699	0.7328

	K_2 (g mg^{-1} min^{-1})	2.3 × 10^{-2}	7.49 × 10^{-3}	9.766 × 10^{-3}
**Pseudo-second order**	Q_e – cal (mg/g)	79.36	104.17	84.03
	Q_e – exp (mg/g)	78.61	97.94	78.78
	R^2	0.999	0.9979	0.9988
**Intraparticle diffusion model**	K_{id} (min$^{0.5}$)	4.31	11.15	9.50
	R^2	0.928	0.9582	0.9151

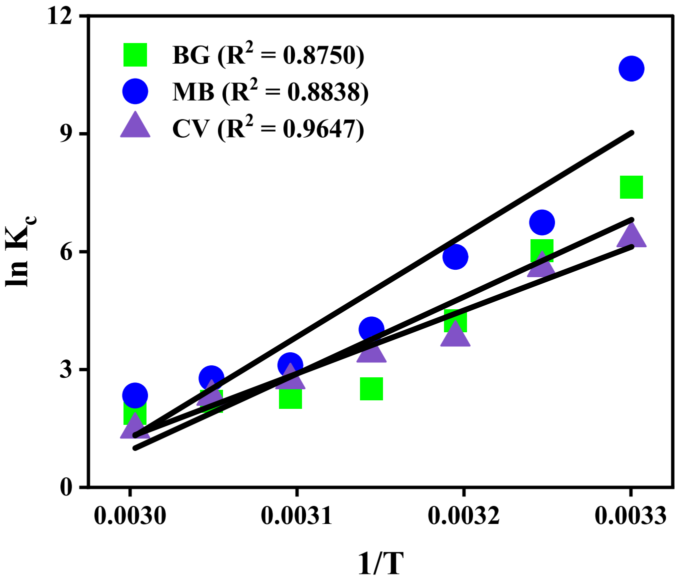

Figure S9. Thermodynamic analysis through Van't Hoff plotting for the adsorption of all dyes onto the PNAC-800 material.

Table S3. Thermodynamic parameter values of Gibbs free energy, enthalpy, and entropy.

**T (K)**	**Methylene blue**			**Brilliant green**			**Crystal violet**		
	**ΔG° (kJ.mol-**	**ΔH° (kJ.mol-**	**ΔS° (kJ.mol-**	**ΔG° (kJ.mol-**	**ΔH° (kJ.mol-**	**ΔS° (kJ.mol-**	**ΔG° (kJ.mol-**	**ΔH° (kJ.mol-**	**ΔS° (kJ.mol-**

	1)	1)	1.K-1)	1)	1)	1.K-1)	1)	1)	1.K-1)
**303**	-2.66			-1.91			-1.58		
**308**	-1.68			-1.50			-1.39		
**313**	-1.46			-1.06			-0.95		
**318**	-1.00	215.66	0.64	-0.62	162.68	0.48	-0.85	133.85	0.39
**323**	-0.77			-0.57			-0.68		
**328**	-0.69			-0.55			-0.58		
**333**	-0.58			-0.47			-0.37		

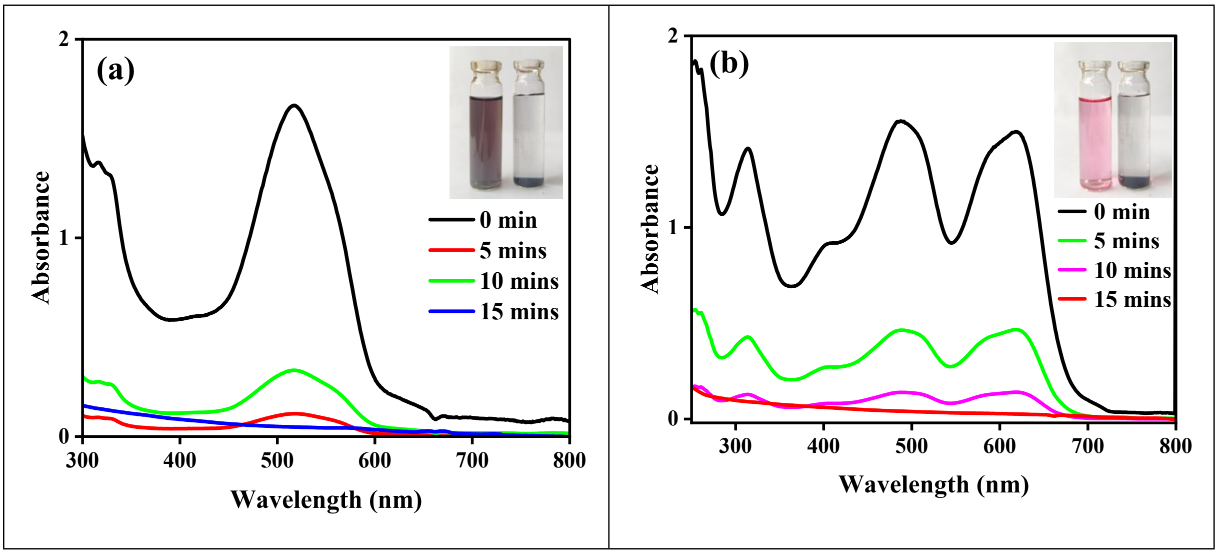

Figure S10. UV-vis spectra depicting the adsorption behaviour in the presence of the synthesized adsorbent, PNAC-800, of (a) black industrial dye effluent, and (b) pink industrial dye effluent.

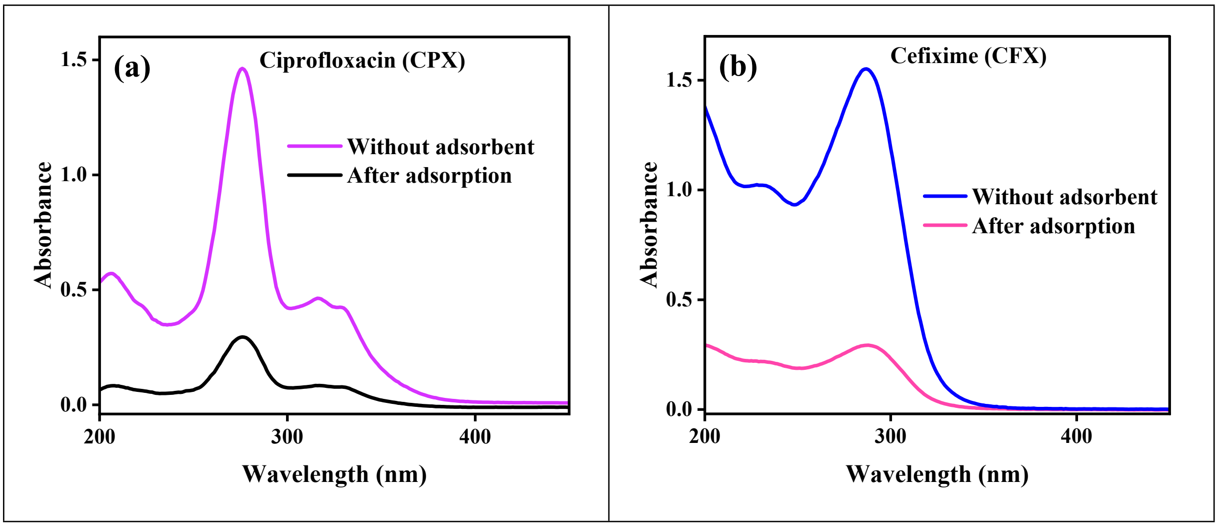

Figure S11. UV-vis spectra illustrating adsorption for (a) Ciprofloxacin and (b) Cefixime using PNAC-800 adsorbent.

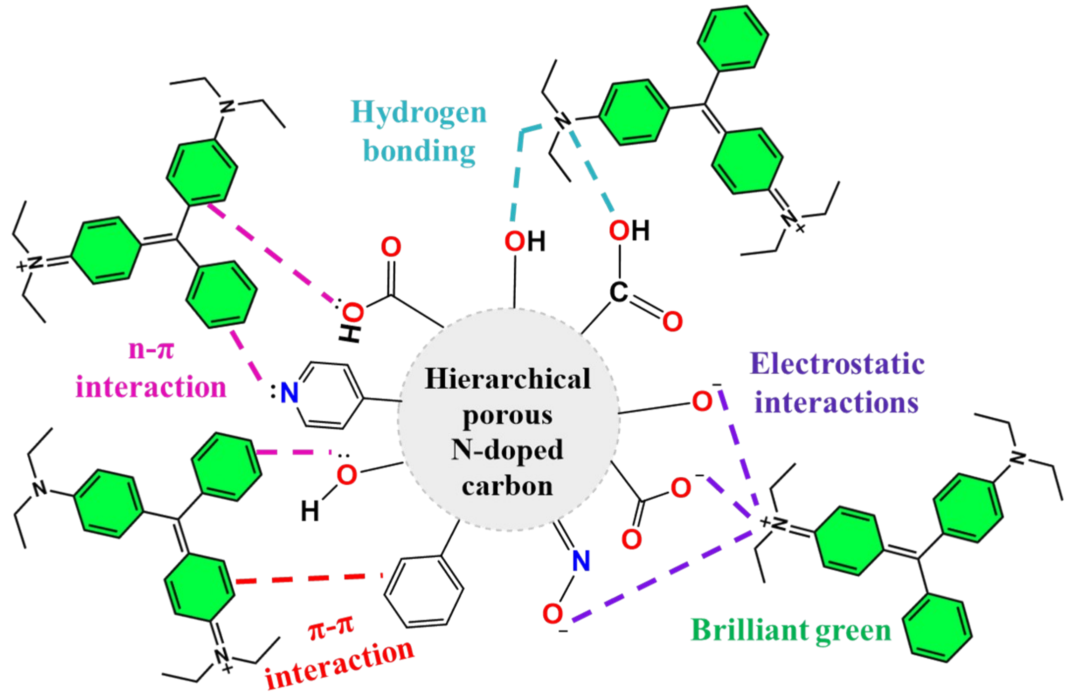

Figure S12. Plausible adsorption mechanism of BG onto the surface of PNAC-800 adsorbent.

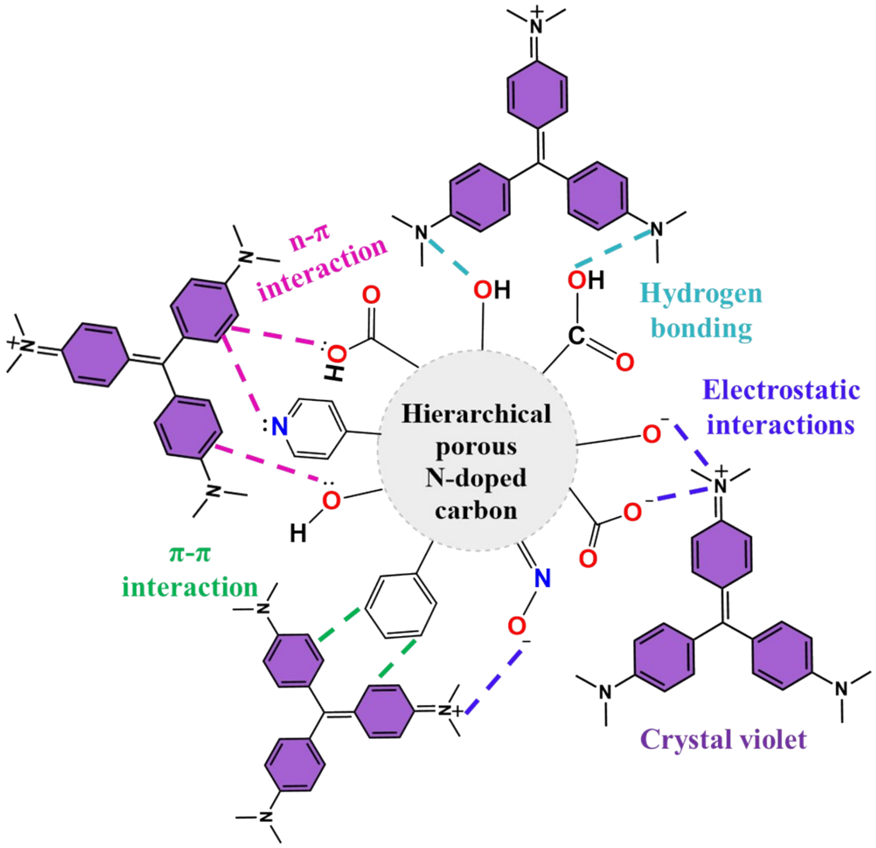

Figure S13. Plausible adsorption mechanism of CV onto the surface of PNAC-800 adsorbent.

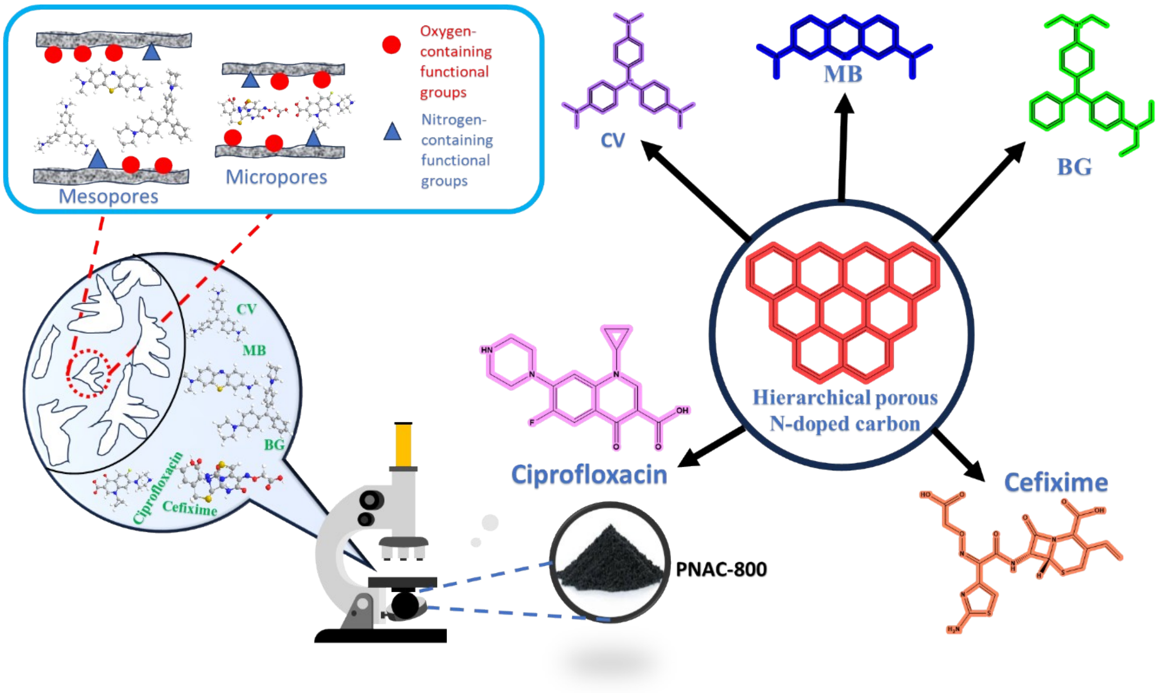

Figure S14. Plausible way of adsorption of dyes (BG, MB, and CV) and pharmaceutical drugs (CPX and CFX) onto PNAC-800 sample by pore filling process.
